# Population-Wide Genetic Risk Prediction of Complex Diseases: A Pilot Feasibility Study in Macau Population for Precision Public Healthcare Planning

**DOI:** 10.1038/s41598-017-19017-y

**Published:** 2018-01-30

**Authors:** Nancy B. Y. Tsui, Gregory Cheng, Teresa Chung, Christopher W. K. Lam, Anita Yee, Peter K. C. Chung, Tsz-Ki Kwan, Elaine Ko, Daihai He, Wing-Tak Wong, Johnson Y. N. Lau, Lok Ting Lau, Manson Fok

**Affiliations:** 1 Department of Applied Biology and Chemical Technology, The Hong Kong Polytechnic University, Hung Hom, Hong Kong; 2 Avalon Genomics (Hong Kong) Limited, Shatin, Hong Kong; 3 Faculty of Health Sciences, Macau University of Science and Technology, Taipa, Macau; 4 Department of Applied Mathematics, The Hong Kong Polytechnic University, Hung Hom, Hong Kong

## Abstract

The genetic bases of many common diseases have been identified through genome-wide association studies in the past decade. However, the application of this approach on public healthcare planning has not been well established. Using Macau with population of around 650,000 as a basis, we conducted a pilot study to evaluate the feasibility of population genomic research and its potential on public health decisions. By performing genome-wide SNP genotyping of over a thousand Macau individuals, we evaluated the population genetic risk profiles of 47 non-communicable diseases and traits, as well as two traits associated with influenza infection. We found that for most of the diseases, the genetic risks of Macau population were different from those of Caucasian, but with similar profile with mainland Chinese. We also identified a panel of diseases that Macau population may have a high or elevated genetic risks. This pilot study showed that (1) population genomic study is feasible in Asian regions like Macau; (2) Macau may have different profile of population-based genetic risks than Caucasians, (3) the different prevalence of genetic risk profile indicates the importance of Asian-specific studies for Asian populations; and (4) the results generated may have an impact for going forward healthcare planning.

## Introduction

Precision medicine is an anticipated goal of human genetics that translates genetic research into medical practice. Precision medicine initially focused on the tailoring of disease treatment according one’s genetic background^1^. This approach has already been used clinically for diagnosis and therapeutic decision making of diseases like cancers^2,3^.

The next important prospect of precision medicine would be towards disease prediction and prevention, before diseases manifest themselves. Genetic risk prediction of common diseases is mostly based on single nucleotide polymorphism (SNP) markers that are associated with disease susceptibility^4,5^. Thus far, more than 24,218 SNP-disease associations have been identified across the genome by genome-wide association studies (GWAS)^6^. Since the risk prediction of most common diseases are complexed by genetic heterogeneity, low penetrance and gene-environment interactions^7^, this approach has not yet been adopted in the clinical setting^8^. Despite the current limitations, genetic risk prediction has gained broad attention in general public due to the availability of direct-to-customer genetic testing products in the markets^9,10^. Along with the rapid advancement of DNA analysing technologies such as next-generation sequencing and microarrays, the cost of genomic testing is lowering steadily, making it affordable and feasible for the general public to have their genomes sequenced and analysed for disease risk profiles. In the near future, it is very likely that preventive measures for high-risk diseases may be devised to improve the overall health planning both for individuals and for healthcare delivery planning.

The precision medicine approach of disease prediction and prevention can be expanded to population-wide level, i.e., precision public healthcare planning^11–13^. In order to implement an effective public healthcare planning for the future in any community/society, genetic data that can assess the present and future disease burdens would be extremely helpful. Population genomics is a potentially very useful tool for evaluating the genetic risk burden of diseases predisposed in a society. This approach has not been firmly established yet because (1) this technology has only been available recently; (2) the number of sampled individuals has to be large enough to adequately represent the population assessed; (3) public databases such as 1000 Genomes Project (1KG)^14^ only contain a few hundred samples each from limited geographic regions; and (4) genomics is a rapidly evolving field that non-genetic healthcare professionals and policy makers may not appreciate the value of this approach in the planning of future healthcare delivery^15^. An in-depth collaboration among geneticists, healthcare scientists and policy makers is critical to address the value of this approach.

The aim of the present study was to determine the feasibility of such a precision public healthcare genetic disease burden assessment in Macau, a society with around 650,000 people^16^, as a pilot test for identifying the challenges and assessing the potential value for other Asian societies. With a population of around 650,000 people in Macau, of which Southern Han Chinese constitute 92.4%^17^, genetic profiling of a thousand Han individuals would represent 0.15% of the total population, and an even higher representation of the future disease burden from a genetic assessment perspective. Owing to the relatively simple healthcare system in Macau, we have also attempted to make some preliminary recommendations for the Macau health authority’s consideration for possible public healthcare planning.

## Results

### Subjects

The genome-wide SNP profiles of 1,366 individuals recruited from the Macau population were obtained. Among the recruited subjects, 1,308 (95.8%) comprised the youth population attending Macau University of Science and Technology (MUST) with an average age of 20.6 years. According to a local census in year 2016, the enrolment rate of higher education in Macau was 79.9%^18^. Hence, the university students recruited in this study were reasonably representative to the Macau youth population. The socioeconomic status of the subjects was not documented. The study was less likely biased toward healthier individuals because for most of the diseases/traits investigated in this study, the symptoms seldom manifest during youth age.

Sixty-one samples were next excluded because the subjects were either non-Chinese (4 samples), non-Han Chinese (22 samples), or genotyping data failed to pass QC (35 samples). This resulted in 1,305 Macau Han Chinese samples which were included in subsequent analysis. In order to reduce ethnic variation due to recent immigrants, we then grouped 1,024 of the samples that were collected from Macau residents born in Macau. They were referred as Macau population in this study (Table 1). These samples represent 2.2% of the general Macau population aged 22–24 years old^18^. The remaining 281 samples were collected from subjects studying or working in Macau (Table 1).

**Table 1:** Information of samples included in the genetic risk analysis.

Population		N (male/female)	Age, average (range)
Macau samples (Han Chinese)	Macau-born residents^1^	1024 (527/497)	21 (17–69)
	Mainland Chinese	281 (128/153)	20 (16–65)
1000 Genomes Project samples	CHS	105 (52/53)	—
	CHB	103 (46/57)	—
	EUR	503 (240/263)	—
	AMR	347 (170/177)	—
Mainland Chinese samples (samples combined from mainland Chinese samples collected in Macau, CHS and CHB)		489 (226/263)	—

^1^The Macau-born residents were considered as Macau population in this study.

### Macau population structure

We compared the population structures of Macau with those in the 1000 Genomes Project (1KG). By using principal component analysis (PCA), the Macau samples were clustered together with the EAS (East Asian) super-population of 1KG as expected (Supplementary Fig. S1).

**Figure 1:**
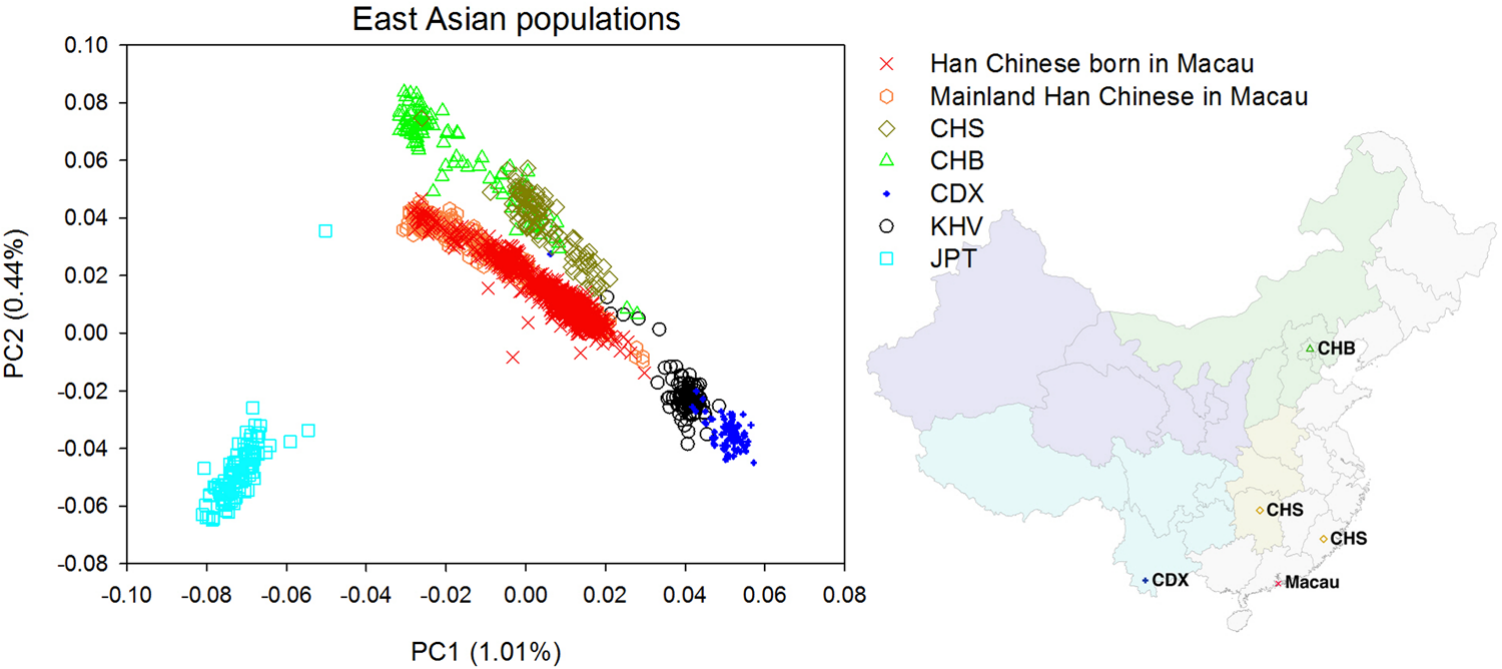
Comparison of population genetic structures among Macau and EAS populations from 1000 Genomes Project. The EAS (East Asian) populations of CHS (Southern Han Chinese), CHB (Beijing Han Chinese), CDX (Chinese Dai in Xishuangbanna), KHV (Vietnamese) and JPT (Japanese) were compared against the Macau samples by PCA. The geographic locations of the Chinese populations are also shown. The map image was modified from Chen *et al*.^37^.

PCA was then performed among the five EAS populations of 1KG (CHS, CHB, CDX, KHV and JPT), as well as the Macau population and the mainland Chinese samples collected in Macau. We found that both groups of the samples collected in Macau tended to distinct from the 1KG Han Chinese populations such as CHS (Southern Han Chinese) and CHB (Beijing Han Chinese) (Fig. 1). This result was not surprising because the population structures of Han Chinese are varied with a north-south geographic stratification^19,20^. For the mainland Chinese subjects recruited in Macau, a majority of them usually reside in Guangdong province of China. Macau and Guangdong are geographically located more southernmost than Hunan and Fujian provinces where CHS samples were collected (Fig. 1). The dissimilar population structures highlighted the need of a “Southernmost Han Chinese” genomic database for more accurate genetic association and population genetic studies.

### Genetic risk profile differences between Macau and Caucasian populations

We investigated the population-wide genetic risks of 47 complex diseases and traits, which included cancers, gastrointestinal, renal, heart and vascular, metabolic, neurological and psychiatric, autoimmune and other diseases (Supplementary Table S1). The diseases were selected because their SNP-disease associations have been demonstrated in the Chinese or East Asian populations. For 38 of the 47 diseases, the SNP-disease associations were also reported in Caucasians (Supplementary Table S1a). Hence in this part of the study, we compared the genetic risks of these 38 diseases between Macau and the Caucasian populations of EUR and AMR from 1KG. Mainland Chinese samples, which were combined from CHS, CHB and our mainland Chinese samples collected in Macau (Table 1), were also included for comparison.

As shown in Table 2, the population genetic risk profiles were significantly different among Macau, mainland Chinese, EUR and AMR for 37 of the 38 diseases (97.4%, *P* < 0.05, Kruskal-Wallis tests for diseases with multiple SNP markers, χ^2^ tests for diseases with single SNP marker). Subsequent pairwise comparisons revealed that the risk differences were mainly due to the significant different risk profiles between Macau and Caucasians (EUR and/or AMR) (*P* < 0.05, Dunn’s pairwise comparisons, Table 2). The Macau population tended to have higher genetic risks than both EUR and AMR for 12 diseases (Table 2). On the other hand, the genetic risks of Macau and mainland Chinese were mostly similar with only 7 out of the 37 diseases showed significant differences in pairwise comparisons (Table 2).

**Table 2:** Statistical comparison of genetic risks among Macau, mainland Chinese, EUR and AMR populations.

Herita-bility	Diseases	*P* (Kruskal-Wallis test)	*P* (Post hoc pairwise comparison, Dunn’s test)		
			Macau vs EUR	Macau vs AMR	Macau vs mainland Chinese^2^
60–80%	Alzheimer’s disease	0.004^1^	<0.05^1^ (Macau < EUR)	not significant^1^	not significant^1^
	Asthma	0.001	≤0.001 (Macau < EUR)	≤0.001 (Macau < AMR)	1.000
	Bipolar disorder	≤0.001	≤0.001 (Macau > EUR)	≤0.001 (Macau < AMR)	1.000
	Hypercholesterolemia	≤0.001	≤0.001 (Macau < EUR)	≤0.001 (Macau < AMR)	1.000
	Psoriasis	≤0.001	≤0.001 (Macau < EUR)	≤0.001 (Macau < AMR)	0.008 (Macau < Chinese)
	Rheumatoid arthritis	≤0.001	≤0.001 (Macau > EUR)	0.273	1.000
	Schizophrenia	≤0.001	≤0.001 (Macau < EUR)	≤0.001 (Macau < AMR)	1.000
	Systemic lupus erythematosus	≤0.001	≤0.001 (Macau > EUR)	0.005 (Macau > AMR)	0.001 (Macau < Chinese)
	Ulcerative colitis	≤0.001	0.948	≤0.001 (Macau > AMR)	1.000
40–60%	Age-related macular degeneration	≤0.001	≤0.001 (Macau > EUR)	≤0.001 (Macau > AMR)	0.003 (Macau > Chinese)
	Cerebral aneurysms	≤0.001	≤0.001 (Macau > EUR)	≤0.001 (Macau < AMR)	1.000
	Coronary heart disease	0.002	1.000	0.003 (Macau > AMR)	1.000
	Crohn’s disease	≤0.001	≤0.001 (Macau > EUR)	≤0.001 (Macau > AMR)	0.197
	Glaucoma	≤0.001	0.020 (Macau > EUR)	≤0.001 (Macau > AMR)	1.000
	Hypertriglyceridemia	≤0.001^1^	not significant^1^	< 0.05^1^ (Macau > AMR)	not significant^1^
	Kidney stones	≤0.001	≤0.001 (Macau > EUR)	≤0.001 (Macau > AMR)	1.000
	Migraine	≤0.001	≤0.001 (Macau < EUR)	≤0.001 (Macau < AMR)	0.259
	Myocardial infraction	≤0.001	≤0.001 (Macau > EUR)	≤0.001 (Macau > AMR)	0.360
	Obesity	0.004	0.534	0.297	0.371
	Primary biliary cholangitis	≤0.001	1.000	≤0.001 (Macau < AMR)	0.789
	Prostate cancer	≤0.001	≤0.001 (Macau < EUR)	≤0.001 (Macau < AMR)	1.000
	Thyroid cancer	0.037	0.542	0.038 (Macau > AMR)	1.000
20–40%	Bladder cancer	≤0.001	≤0.001 (Macau < EUR)	1.000	1.000
	Breast cancer	0.207	—	—	—
	Colorectal cancer	≤0.001	0.459	≤0.001 (Macau > AMR)	1.000
	Endometrial cancer	≤0.001	≤0.001 (Macau > EUR)	≤0.001 (Macau > AMR)	1.000
	Gallstone	≤0.001^1^	< 0.05^1^ (Macau > EUR)	< 0.05^1^ (Macau > AMR)	not significant^1^
	Glioma	≤0.001	≤0.001 (Macau < EUR)	≤0.001 (Macau < AMR)	1.000
	Hypertension	≤0.001	≤0.001 (Macau < EUR)	≤0.001 (Macau < AMR)	0.044 (Macau < Chinese)
	Kidney cancer	≤0.001	0.259	≤0.001 (Macau < AMR)	≤0.001 (Macau > Chinese)
	Ovarian cancer	0.003	0.019 (Macau < EUR)	1.000	0.742
	Pancreatic cancer	≤0.001	≤0.001 (Macau < EUR)	≤0.001 (Macau < AMR)	1.000
	Parkinson’s disease	≤0.001	≤0.001 (Macau > EUR)	≤0.001 (Macau > AMR)	< 0.05 (Macau > Chinese)
	Stroke	≤0.001	≤0.001 (Macau > EUR)	≤0.001 (Macau > AMR)	1.000
	Type 2 diabetes	≤0.001	≤0.001 (Macau > EUR)	≤0.001 (Macau > AMR)	0.037 (Macau < Chinese)
10–20%	Lung cancer	≤0.001	≤0.001 (Macau > EUR)	≤0.001 (Macau > AMR)	1.000
	Non-Hodgkin lymphoma	≤0.001	≤0.001 (Macau < EUR)	≤0.001 (Macau < AMR)	0.516
Unknown	Narcolepsy	≤0.001	≤0.001 (Macau > EUR)	0.014 (Macau < AMR)	1.000

Diseases with *P* < 0.05 were considered as having significant differences. ^1^χ^2^ test was performed since only one SNP marker was utilised for risk prediction. Ransacking approach was used for post-hoc pairwise comparison. Z value was calculated and compared against the square root of χ^2^ critical value with 3 degrees of freedom of 2.8. ^2^Mainland Chinese refers to the combined samples of CHS, CHB, and mainland Chinese individuals recruited from Macau.

**Figure 2:**
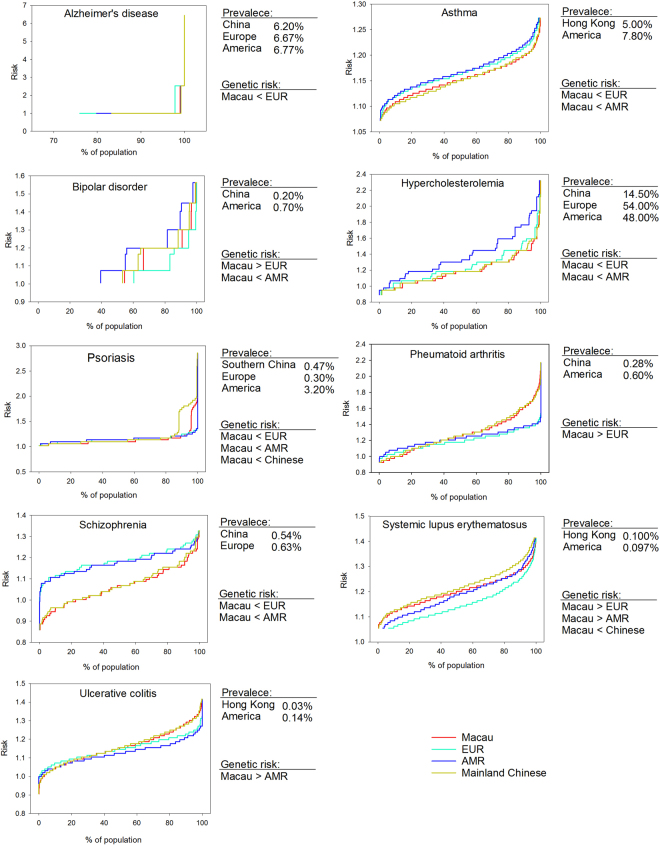
Comparisons of population-wide genetic risk distributions among Macau, mainland Chinese, EUR and AMR. Population-wide cumulative distributions of genetic risks are plotted against percentages of populations. Diseases with heritability of 60–80% and with significant differences of genetic risks among the populations are shown. Disease prevalence and the result of genetic risk comparison are tableted next to each plot of the disease.

We next investigated if the results of our genetic risk prediction could be revealed by the observed disease prevalence of the populations. The risks of developing complex diseases are in fact influenced by genetic factors in different extents, i.e., different heritability^21^. We reasoned that the higher the heritability of a disease, the more likely its genetic risk would be reflected by the disease prevalence due to the less influence by environmental factors. We therefore focused on nine diseases that had the highest heritability of 60–80%, and compared their trends of genetic risk differences among the populations with the prevalence in the corresponding regions or regions nearby (Supplementary Fig. S2). As shown in Fig. 2, 7 of the 9 diseases tended to have matched trends between the population genetic risks and the disease prevalence. Hence, the results support the validity of our genetic risk prediction.

The pattern of genetic risk distribution could act as another parameter for investigating disease burden of a population. For example, for psoriasis, although the overall genetic risk of Macau was lower than that of EUR and AMR (Table 2), we observed a small group of Macau individuals who tended to have unusually higher genetic risks when compared to the rest of the population (Fig. 2). Such population risk distribution may have an impact on healthcare planning for the indicated society.

### Genetic risk categorisation of Macau population

To facilitate the interpretation of the genetic risk result, for each disease, we categorised the Macau population samples into “high”, “elevated”, “average” and “reduced” genetic risks by REGENT^22,23^. A total of 47 complex diseases with reported SNP-disease associations in Chinese or East Asian populations (Supplementary Tables S1a and b) were analysed by REGENT. The categorisations of 35 diseases showed area under curves of >0.6 (Supplementary Table S2), and were included for further analysis.

**Figure 3:**
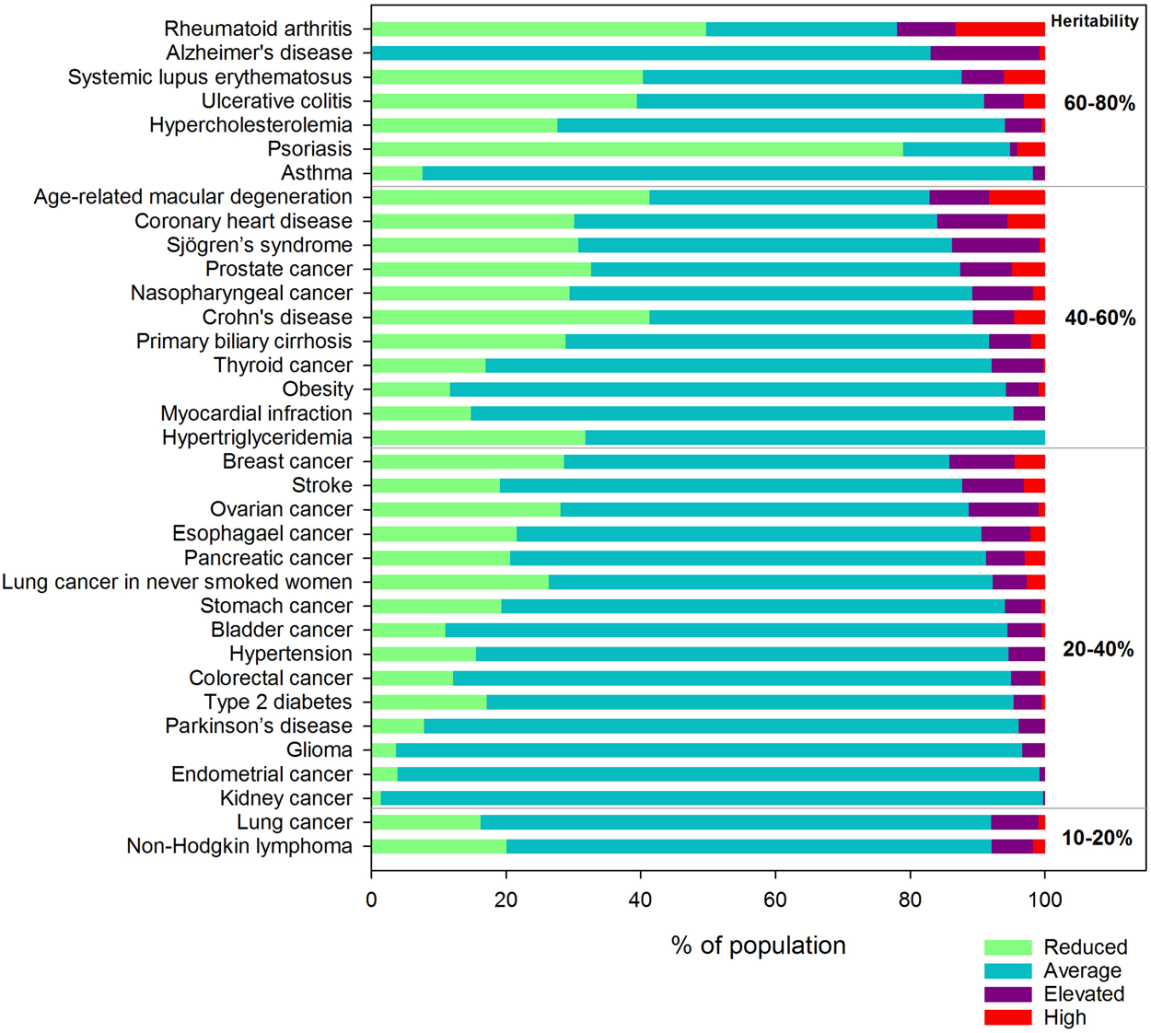
Proportions of Macau population that had reduced, average, elevated and high genetic risks of the diseases.

Figure 3 shows the proportions of Macau population that had high, elevated, average and low genetic risks for the 35 diseases. Among them, we considered 12 diseases to be important to Macau because over 10% of the population had high or elevated genetic risks of the diseases. These diseases were rheumatoid arthritis (22.0%), age-related macular degeneration (17.1%), Alzheimer’s disease (17.0%), coronary heart disease (16.0%), breast cancer (14.2%), Sjögren’s syndrome (13.8%), prostate cancer (12.6%), systematic lupus erythematosus (SLE) (12.4%), stroke (12.3%), ovarian cancer (11.3%), nasopharyngeal cancer (10.8%) and Crohn’s disease (10.7%).

### Genetic traits in response to influenza infection

One very important concern of public healthcare in Macau, a densely-populated city, is infectious diseases. We therefore investigated the feasibility of predicting the impact of an infectious disease based on the genetic background of the society. We chose influenza as an illustrative example because the disease is pandemic in Southern China including Macau annually. Two traits associated with influenza infection were studied. They were the severity of illness after influenza A (H1N1pdm09) infection^24,25^ (personal communication with Prof K. Y. Yuen, manuscript submitted), and avian influenza (H7N9) susceptibility^25,26^ (Supplementary Table S1c).

**Figure 4:**
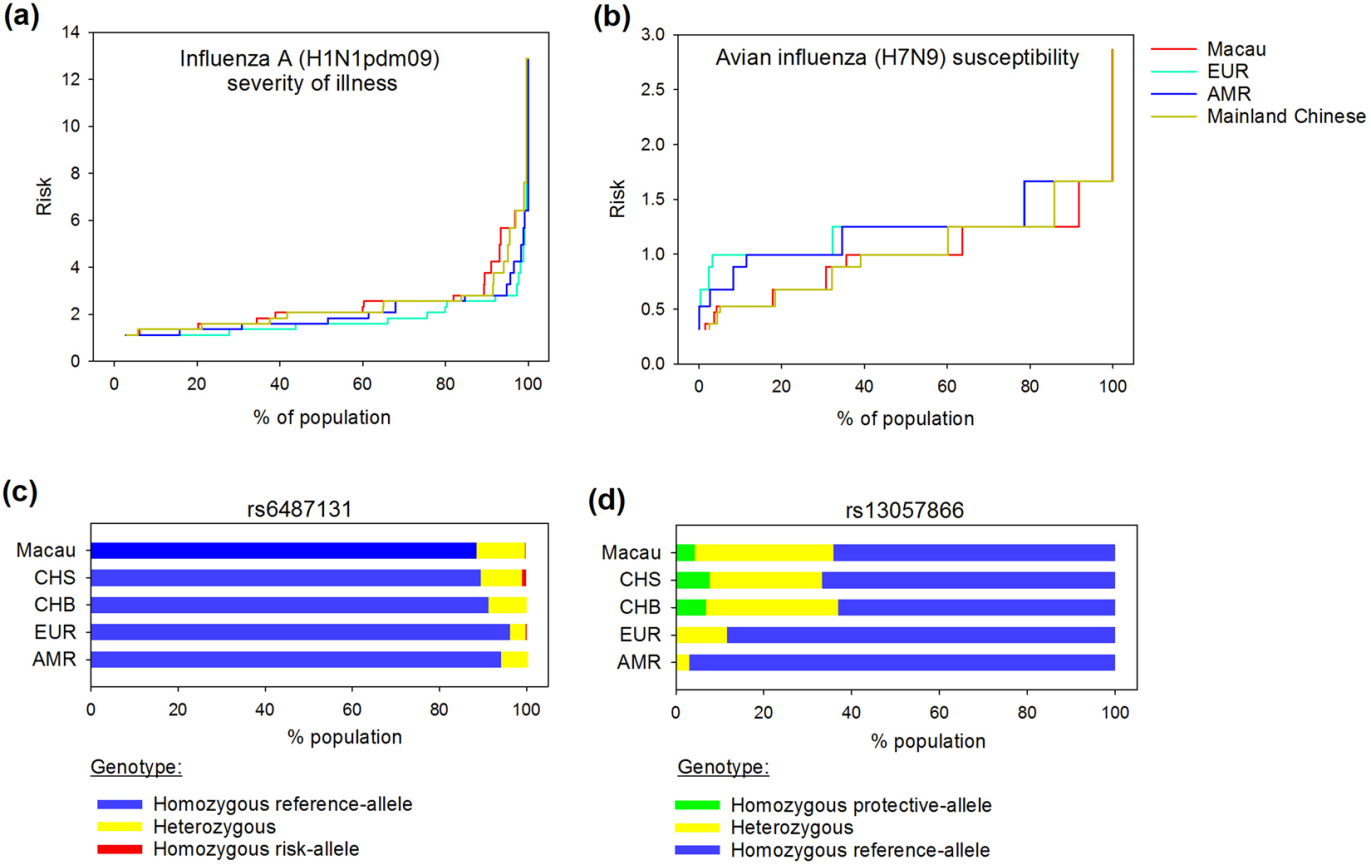
Comparisons of population genetic risks of traits associated with influenza infection among Macau, mainland Chinese, EUR and AMR. Population-wide cumulative distributions of the genetic risks of (**a**) influenza A (H1N1pdm09) disease severity and (**b**) avian influenza (H7N9) susceptibility. Population frequencies of (**c**) the risk-allele of rs6487131 and (**d**) the protective-allele of rs13057866 in Macau, CHS, CHB, EUR and AMR.

For H1N1pdm09 influenza, Macau population had a significantly higher genetic risk of having severe illness after infection, when compared to EUR and AMR (*P* < 0.05, Kruskal-Wallis tests and Dunn’s pairwise comparisons) (Table 3, Fig. 4a). Owing to the small sample size of the SNP discovery study (personal communication with Prof K. Y. Yuen, manuscript submitted), REGENT was unable to categorise the population genetic risks. We therefore looked into one of the SNP markers, rs6487131, which had the largest effect size with odds ratio (OR) of 16.6 (Supplementary Table S1c and Supplementary Fig. S4c). We found that 11.3% of the Macau population carried at least one copy of the risk allele of rs6487131, hence may be at a higher risk of developing severe illness when compared to the rest of the population. In fact, when we inspected the risk-allele frequencies among Macau, CHS and CHB, we found an increasing trend of the abundance of the risk-allele from the northern to the southern regions of China (Fig. 4c).

**Table 3:** Statistical comparison of the genetic risks of traits associated with influenza infection among Macau, mainland China, EUR and AMR populations.

Traits	*P* (Kruskal-Wallis test)	*P* (Post hoc pairwise comparison, Dunn’s test)		
		Macau vs EUR	Macau vs AMR	Macau vs mainland Chinese
Influenza A (H1N1pdm09) severity of illness	≤0.001	≤0.001 (Macau > EUR)	≤0.001 (Macau > AMR)	1.000
Avian influenza (H7N9) susceptibility	≤0.001	≤0.001 (Macau < EUR)	≤0.001 (Macau < AMR)	1.000

Traits with *P* < 0.05 were considered as having significant differences.

For H7N9 influenza, EUR and AMR both had a significantly higher genetic susceptibility than Macau (*P* < 0.05, Kruskal-Wallis tests and Dunn’s pairwise comparisons) (Table 3, Fig. 4b). The protective allele of rs13057866, which has an OR of 0.29 (Supplementary Table S1c and Supplementary Fig. S4c), was relatively common in Macau with 35.8% of the Macau population carried at least one copy of the allele (Fig. 4d). In contrast, only 11.6% and 3.0% of EUR and AMR populations, respectively, carried the protective allele (Fig. 4d).

## Discussion

The ultimate goal of precision public healthcare is to be able to set up a plan to prevent disease, promote health and reduce health disparities in a population^12,13^. Despite the ongoing debate on its benefit to improve population health^27^, technological advances in genomics research make this discipline evolving into an important possible component for future public healthcare planning. This pilot study has demonstrated the feasibility and the potential utility of this approach in helping future healthcare planning for health authorities.

**Figure 5:**
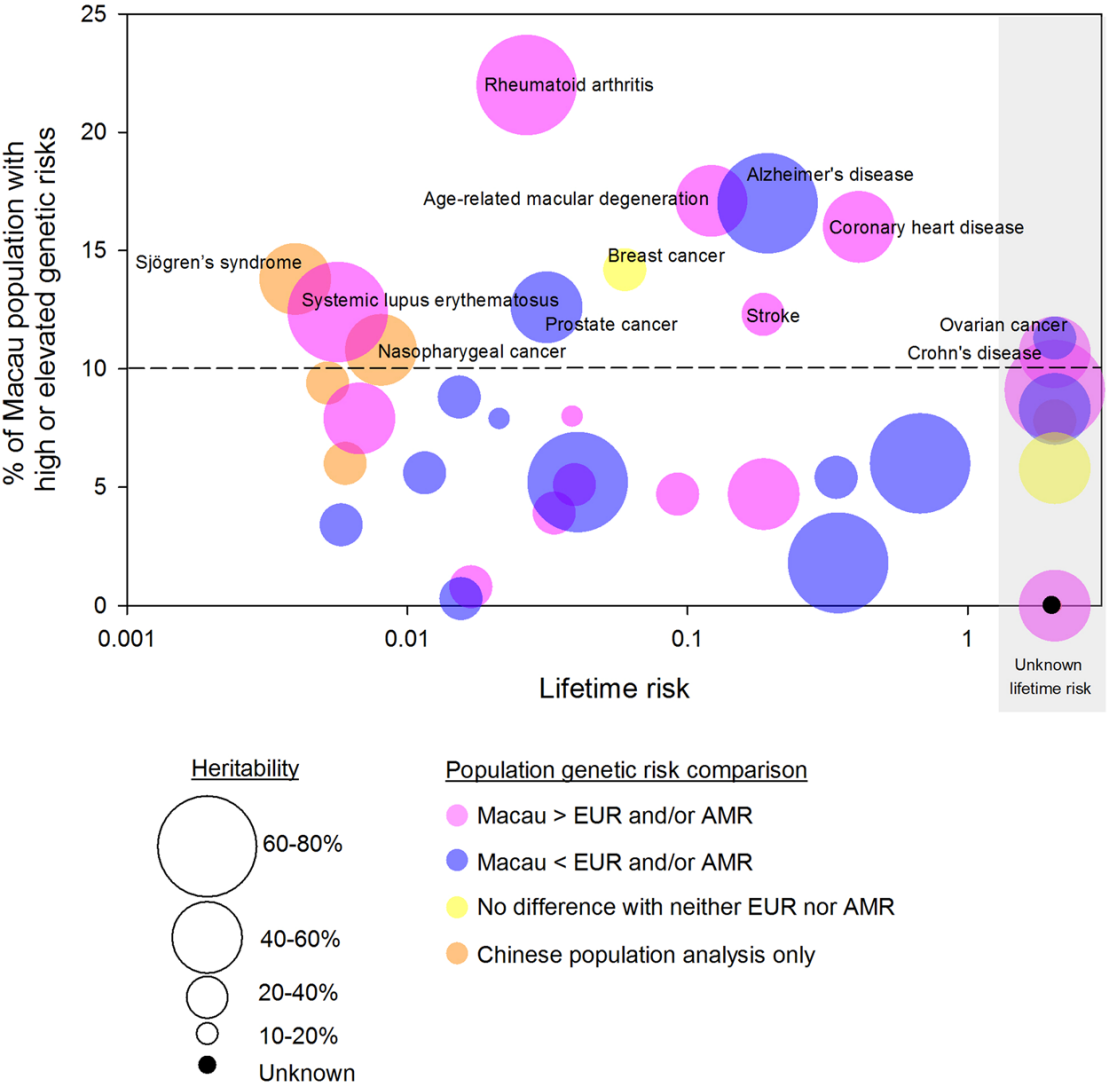
Genetic risk characteristics of the studied diseases in Macau population. For each disease, the percentage of Macau population having high/elevated genetic risk is plotted against lifetime risk. The lifetime risks were available for 30 of the 37 risk-categorised diseases. Twelve diseases with more than 10% of Macau population having high/elevated genetic risks were labelled in the graph. The population risks of Macau were higher than that of EUR and/or AMR for six of the labelled diseases (red bubbles). Heritability is represented by the bubble size.

Figure 5 summarises the findings of this study. We consider a disease to be important to the Macau population based on the following factors:

### Diseases with a large proportion of population predisposing relatively high genetic risks

There were 12 complex diseases in which more than 10% of the Macau population having high/elevated genetic risks. These diseases are labelled in Fig. 5. Rheumatoid arthritis would be of particular concern because more than one-fifth of the Macau population would be at relatively high genetic risk.

The relatively abundant genetically-at-risk population of these diseases, especially rheumatoid arthritis, suggests that population-level interventions targeting these diseases should be considered by the government and healthcare organisations in order to lower the overall risks of diseases manifestation. Examples of healthcare planning include public resource allocation, healthcare personnel training, promotion of the awareness of the diseases by public, launching of health promotion programs in the aspects of lifestyle, nutritional and social, and tackling of environmental hazards.

### Diseases with high lifetime risks

Coronary heart disease, Alzheimer’s disease, stroke and age-related macular degeneration have the highest lifetime risks of greater than 0.1 among the other diseases in Fig. 5. When focusing on cancer, the top cause of death in Macau^28^, the lifetime risks of breast cancer and prostate cancer were greater than 0.01. The high lifetime risks together with the relatively high proportions of genetically-at-risk population of these diseases would made them equally important as rheumatoid arthritis for the Macau population. This will again provide input with regards to future healthcare planning in Macau.

### Diseases specifically important for Macau population but not Caucasians

When compared to EUR and/or AMR, Macau has significantly higher genetic risks of rheumatoid arthritis, age-related macular degeneration, coronary heart disease, SLE, stroke and Crohn’s disease among the diseases in Fig. 5. In addition, Sjögren’s syndrome and nasopharyngeal cancer from Fig. 5 are locally important diseases as evidence by the SNP-disease association studies reported in Chinese but not European and American. We suggested that instead of referencing the healthcare programs and policies from Western societies, a more Macau-oriented public healthcare planning should be considered for these diseases. The findings also highlight the importance of population focus/specific studies for the healthcare planning and not just referring to the literature with data derived from other populations.

### Effect sizes of genetics on overall disease risks

The higher the heritability of a disease, the less likely the disease risk could be modified by environmental factors such as lifestyle. In fact, rheumatoid arthritis, Alzheimer’s disease and SLE have the highest heritability implications of over 60% among the diseases in Fig. 5. This suggests that the risks of these diseases would be relatively difficult to modify. Hence, in addition to the interventions suggested above population-wide screening programs for early detection of these diseases in the population should also be considered.

### Pandemic preparation of infectious diseases

We found that Macau population tended to have a higher genetic risk of developing severe illness after H1N1pdm09 viral infection, when compared to the populations in the rest of China as well as Caucasians. Hence, in addition to health promotion, emergency preparation of the pandemic would be important for Macau. Furthermore, Macau has a huge tourist population. Travel advice or measures may be considered for tourists from Western countries such as Europe and America, whom may have a higher genetic susceptibility of H7N9 influenza than the local Macau population.

Genetically contributed disease risks, which are more stable than environmental risks, would provide valuable information for the health authorities to plan for long-term healthcare delivery. In fact, an increasing amount of studies have revealed an important role of genetic factors in understanding population disease risks. For example, Haiman *et al*. have reported an ethnic difference in the association of cigarette smoking and lung cancer risk. However, this difference could not be fully explained by factors including diet, occupation and socioeconomic status^29^. Similarly, Ollberding *et al*. have reported that Japanese Americans and African American women were at higher risk of colorectal cancer than whites in US, but extrinsic factors, such as BMI, cigarette smoking and dietary risk factors, could not fully account for the ethnic disparity^30^. These studies suggest that in order to comprehensively assess the disease risks of a population, genetic risks would be a valuable add-on factor. They are particularly useful in regions, such as Macau, where epidemiological statistics of a large variety of diseases are not always available.

This study would serve as the first step of precision public healthcare planning. We have predicted the genetic susceptibility of a panel of common diseases and identify potential health burden of Macau. The next important step should be the adoption of the findings by the government and public healthcare organisations of Macau to consider planning and also potential policy implications. Our findings may assist the planning of targeted prevention and treatment strategies such that public resources could be effectively directed to diseases important to Macau.

To allow better assessment of Macau population health, two essential components should be continued to build up. The first component is a locality-specific Macau population genomic database. In this study, we have initiated this database by surveying genome-wide SNP profiles of more than a thousand Macau individuals. Youth population was firstly targeted because they will be the key labour force of Macau in the future, and hence long-term healthcare planning addressing their risks would be worthwhile. For the next step, we shall enlarge the database and include more diverse Macau sub-populations, such as current adult employee of Macau and non-Macau-born residents, in order to enhance the representation of the database.

The second component is availability of genetic biomarkers that are most relevant to Macau population. The SNP markers used for Macau population in this study and their ORs were selected from a mixture of GWAS based on Chinese, East Asian or Caucasian. In fact, whether the translation of GWAS results from one ethnic group to another may introduce bias and/or noise is still under debate. To estimate if there was systematic bias in applying Caucasian GWAS result in risk calculation of Macau population, we selected 16 of the analysed diseases in which the Chinese- and Caucasian-relevant SNP panels shared ≤50% of similarity, and re-calculated the population risks of the Macau samples using the Caucasian-relevant SNP panels (Supplementary Fig. S3). The Macau population risks became higher for six diseases and lower for ten diseases when compared to the risks calculated using the ethnic-relevant Chinese SNP panels (Supplementary Fig. S3). This observation is not surprising because heterogeneity in disease associations, such as ORs and direction of effects, has been reported between European and Asian^31^. However, in general, we did not observe a systematic trend of under- or over-estimation of population risks among the 16 diseases when the Macau samples were analysed using Caucasian-relevant SNPs (Supplementary Fig. S3). Nonetheless, validation of the SNP markers within Macau population is vitally important for risk prediction accuracy^32^. This, however, requires population genomic data that are sufficiently large and representative. By continual data accumulation of the Macau genomic database, this study would pave the way for future GWAS and other genetic-disease association studies. In fact, Macau may be an ideal location for the testing of this approach.

In conclusion, we have demonstrated the feasibility of a genetic approach for precision public healthcare, and have revealed a panel of diseases that tend to be important for the Macau population. The impact of this finding to public healthcare would highly depend on the well-coordinated efforts among scientists, medical workers and policy makers. The establishment of the Macau genomic database would allow the development of more accurate genetic markers for health assessment at both population and individual levels in Macau.

## Methods

### Subject recruitment

We recruited 1,307 students and 10 staffs from the University Hospital of MUST. Three millilitres of peripheral blood were collected from each subject. Forty-nine subjects from the Macau general public were also recruited, and buccal swab samples were collected. Demographic data were collected through self-reported questionnaires. The study was approved by the Clinical Research Ethics Committee of the University Hospital, MUST. All subjects were recruited with written informed consent. All experiments were performed in accordance with the relevant guidelines and regulations.

### DNA extraction and genome-wide SNP genotyping

DNA was extracted from peripheral blood by QIAamp DNA Blood Mini Kit (Qiagen) and QIAamp 96 DNA Blood Kit (Qiagen) following the manufacturer’s protocols. For buccal swab samples, the foam heads of each sample were soaked in 300 μL PBS. DNA was then extracted by illustra blood genomicPrep Mini Spin Kit (GE Healthcare) according to the manufacturer’s instructions.

All samples were genotyped by Infinium OmniZhongHua-8 BeadChips (Illumina) following the Infinium HD Super Protocol (Illumina). Genotyping Module v2.0 of GenomeStudio (Illumina) was used to call genotypes from raw data. QC procedures were performed by PLINK v1.9^33^. Samples were excluded from subsequent analysis if SNP call rates <95%, heterozygosity <−0.05 or >0.05, or cryptic relatedness >0.1875. Individual SNPs were excluded from analysis if they have missingness >5% across all of the samples, or violated Hardy-Weinberg equilibrium (*P* < 1 × 10^−6^).

### SNP marker search

Disease-associated SNP markers and their ORs were searched from the GWAS database in National Human Genome Research Institute (NHGRI)^6^ and from published studies in PubMed (Supplementary Table S1 and Supplementary Fig. S4). Since many of the GWAS were primarily conducted in Caucasians, especially populations of European descent^32^, we firstly searched for SNP markers that were discovered among European and/or American populations, i.e., “Caucasian-relevant SNP panels”. SNPs that met one of the following criteria were selected: (1) reached genome-wide significance (*P* < 1 × 10^−7^) in GWAS discovery analyses; (2) reached genome-wide association of *P* < 1 × 10^−6^ in GWAS discovery analyses, and significant association (*P* < 0.05) in replication analyses; or (3) significant association (*P* < 0.05) in candidate SNP approach studies. Endometrial cancer was an exception of these criteria because many of the GWAS identified SNPs did not reach the threshold *P*-value of 1 × 10^−7^. Instead, SNPs that reached genome-wide association of *P* < 1 × 10^−5^ were included for endometrial cancer analysis in this study. For SNPs reported in multiple studies, the result from those with the largest sample sizes and/or the most relevant ethical groups were used.

We next transformed the “Caucasian-relevant SNP panels” into “Chinese-relevant SNP panels” with the following modifications: (1) if the SNPs were validated among East Asian, preferably Han Chinese, their ORs were adopted; (2) if the SNPs were investigated in East Asian or Han Chinese but no disease associations were demonstrated, the SNPs were removed from the panels; and (3) SNPs that were discovered among Han Chinese were added.

Finally, for each disease, the Caucasian-relevant SNPs were compared with EUR and AMR haplotypes while the Chinese-relevant SNPs were compared with CHB and CHS haplotypes in IKG database in order to filtered out SNPs with strong linkage disequilibrium (r^2^ < 0.8).

### Principal component analysis (PCA)

PCA was performed with EIGENSTRAT for population stratification^34^. Thinning was first conducted for the data of Macau samples, followed by removing SNPs with high linkage disequilibrium and within non-autosomal regions. This resulted in 126,774 SNPs. PCA was performed using this SNP set for the Macau samples and samples from the populations of 1KG.

### Genetic risk calculation

For each disease and trait, we calculated the genetic risk of each sample based on the polygenic multiplicative OR model that each risk-allele has multiplicative effect on the overall disease susceptibility^35^. We calculated the risk by multiplying the genotype ORs of all SNP markers of the disease, then rooting by the number of the SNPs used, assuming an independent combinatorial effect of the SNPs. The genetic risks were calculated for individual samples of Macau, CHB and CHS populations with the “Chinese-relevant SNP panels” (Supplementary Fig. S4). If population-relevant ORs were unavailable, the ORs of Caucasians were used. The genetic risks of EUR and AMR populations were calculated with the “Caucasian-relevant SNP panels” (Supplementary Fig. S4).

For the traits associated with influenza infection, the SNP markers and their ORs were developed based on Southern Chinese population mainly (Supplementary Table S1c). We used these markers and OR values for calculating the genetic risk of both Chinese (Macau and mainland Chinese) and Caucasian (EUR and AMR).

### Disease risk categorisation

The population-distribution and categorisation of the genetic risks of Macau samples were performed by REGENT (Risk Estimation for Genetic and Environmental Traits)^22,23^. In brief, for each disease, the case-control sample sizes of the corresponding SNP discovery studies, ORs and risk-allele frequencies of the SNP markers (Supplementary Fig. S4) and diseases prevalence (Supplementary Fig. S2) were input into REGENT. The programme then computed the confidence intervals of risk estimates, and allocated the samples into the risk categories of “reduced”, “average”, “elevated” and “high”.

### Statistical analysis

Statistical analysis was conducted using SigmaPlot 13 (Systat Software, Inc). To compare disease risks among populations, Kruskal-Wallis one-way analysis of variance tested were used, with post-hoc pairwise comparisons performed by Dunn’s method. For diseases with one SNP marker, χ^2^ tests were performed with post-hoc analyses carried out using the Ransacking method^36^.

The datasets generated during and/or analysed during the current study are available from the corresponding authors on reasonable request.

## Electronic supplementary material

Supplementary Information
