# Supplementary material for: Population-Wide Genetic Risk Prediction of Complex Diseases: A Pilot Feasibility Study in Macau Population for Precision Public Healthcare Planning
Source: Sci Rep. 2018 Jan 30;8:1853. doi: 10.1038/s41598-017-19017-y (PMC5789865; doi:10.1038/s41598-017-19017-y)
Supplement: Supplementary file 1 — Supplementary Information [file 41598_2017_19017_MOESM1_ESM.pdf]

Supplementary Information

Population-Wide Genetic Risk Prediction of Complex Diseases: A Pilot Feasibility Study in Macau Population for Precision Public Healthcare Planning

Nancy B. Y. Tsui[1,2#], Gregory Cheng[3#], Teresa Chung[1], Christopher W. K. Lam[3], Anita Yee[2], Peter K. C. Chung[2], Tsz-Ki Kwan[2], Elaine Ko[2], Daihai He[4], Wing-Tak Wong[1], Johnson Y. N. Lau[1], Lok-Ting Lau[1]*, Manson Fok[3]*

[1]Department of Applied Biology and Chemical Technology, The Hong Kong Polytechnic University, Hong Kong

[2]Avalon Genomics (Hong Kong) Limited, Hong Kong

[3]Faculty of Health Sciences, Macau University of Science and Technology, Macau

[4]Department of Applied Mathematics, The Hong Kong Polytechnic University, Hong Kong

[#]Co-first authors: Nancy B. Y. Tsui and Gregory Cheng

*Co-corresponding authors: Lok-Ting Lau and Manson Fok

Supplementary Table S1. Diseases investigated in this study and their SNP markers used for genetic risk calculation

Supplementary Table S1a. Diseases in which the population genetic risks among Macau, mainland Chinese, EUR and AMR were compared. SNP markers for genetic risk calculation of the populations of Chinese (Macau and mainland Chinese) and Caucasian (EUR and AMR) are shown.

**Diseases**	**Populations**	**SNP markers**
		Cancers:
Bladder cancer (1-6)	Chinese	rs1014971, rs10936599, rs11543198, rs1495741, rs17674580, rs2294008, rs6104690, rs8102137, rs9642880
	Caucasian	rs1014971, rs10936599, rs11543198, rs1495741, rs17674580, rs2294008, rs6104690, rs8102137, rs9642880
Breast cancer (7-16)	Chinese	rs10069690, rs11249433, rs13387042, rs1562430, rs16886034, rs16886113, rs17530068, rs2284378, rs2363956, rs2981579, rs3112612, rs3757318, rs3803662, rs3822625, rs4700485, rs909116
	Caucasian	rs10069690, rs11249433, rs13387042, rs1562430, rs16886034, rs16886113, rs17530068, rs2284378, rs2363956, rs2981579, rs3112612, rs3757318, rs3803662, rs3822625, rs889312, rs909116
Colorectal cancer (17-26)	Chinese	rs1035209, rs10795668, rs2423279, rs3217901, rs3802842, rs4813802, rs4939827, rs4948317, rs6687758, rs6983267, rs7014346, rs704017, rs7229639
	Caucasian	rs1035209, rs10411210, rs12953717, rs2423279, rs3217901, rs3802842, rs4444235, rs4464148, rs4813802, rs4939827, rs6687758, rs7014346, rs7229639
Endometrial cancer (27-33)	Chinese	rs10757274, rs10887831, rs12514742, rs1777220, rs17782313, rs2891316, rs4680, rs4977756, rs564398, rs5940807, rs6468613, rs6499640, rs6724138, rs673604
	Caucasian	rs10757274, rs10887831, rs11679180, rs12514742, rs12536378, rs12778749, rs1777220, rs2891316, rs4430796, rs4812563, rs4977756, rs564398, rs5940807, rs6468613, rs6724138, rs673604, rs7501939
Glioma (34-37)	Chinese	rs2736100, rs2853676, rs4977756, rs498872, rs6010620
	Caucasian	rs2736100, rs2853676, rs4977756, rs498872, rs6010620
Kidney cancer (38-44)	Chinese	rs1049334, rs1049380, rs10811474, rs1130409, rs1326889, rs1801133, rs2493137, rs3889728, rs405509, rs718314, rs8106922
	Caucasian	rs1049334, rs1049380, rs10811474, rs1326889, rs2493137, rs3889728, rs405509, rs718314, rs8106922
Lung cancer (45-56)	Chinese	rs1051730, rs12296850, rs17576, rs2494938, rs2736100, rs2808630, rs31489, rs402710, rs4809957, rs667282, rs7626795
	Caucasian	rs1051730, rs13314271, rs2736100, rs2808630, rs31489, rs402710, rs4975616, rs7626795
Non-Hodgkin lymphoma (57-66)	Chinese	rs10484561, rs14448, rs1800630, rs1800896, rs1801274, rs1805812, rs2516482, rs2621416, rs3751913, rs4530903, rs4937362, rs4938573, rs4952887, rs6457327, rs6773854, rs702019, rs707824, rs735665, rs9268853
	Caucasian	rs10484561, rs14448, rs1800629, rs1800630, rs1800896, rs1801274, rs1805812, rs2621416, rs3751913, rs4530903, rs4937362, rs4938573, rs4952887, rs6457327, rs6773854, rs702019, rs707824, rs735665, rs9268853
Ovarian cancer (67-71)	Chinese	rs10088218, rs11175194, rs1413299, rs2292730, rs2665390, rs3814113, rs757210, rs7797466
	Caucasian	rs10088218, rs2292730, rs2665390, rs3814113, rs757210, rs7797466
Pancreatic cancer (72-75)	Chinese	rs1486134, rs1517037, rs1547374, rs1561927, rs16986825, rs17688601, rs2255280, rs372883, rs3790844, rs657152, rs6971499, rs7190458, rs7214041, rs9543325, rs9581943
	Caucasian	rs1486134, rs1517037, rs1561927, rs16986825, rs17688601, rs3790844, rs657152, rs6971499, rs7190458, rs7214041, rs9543325, rs9581943
Prostate cancer	Chinese	rs1016343, rs103294, rs10993994, rs11228565, rs12653946, rs1512268, rs17023900, rs1859962, rs2660753, rs339331, rs4242382, rs4430796, rs4962416, rs5759167, rs5945572, rs6983267, rs7127900, rs7130881, rs7931342, rs817826, rs902774, rs9364554, rs9600079

(76-85)	Caucasian	rs1016343, rs10993994, rs11228565, rs1512268, rs17023900, rs1859962, rs2660753, rs4242382, rs4430796, rs4962416, rs5759167, rs5945572, rs6983267, rs7127900, rs7130881, rs7501939, rs7931342, rs902774, rs9364554
Thyroid cancer (86-91)	Chinese	rs1799782, rs861539, rs944289, rs965513, rs966423
	Caucasian	rs11609374, rs1799782, rs944289, rs965513, rs966423
Gastrointestinal and renal diseases:		
Crohn's disease (92-101)	Chinese	rs1004819, rs11175593, rs11195128, rs11235667, rs11805303, rs1893217, rs2076756, rs224136, rs2274471, rs3094188, rs3828309, rs682333, rs6856616, rs7714584, rs7765379, rs7807268, rs7856856, rs9267911, rs9271366, rs9292777, rs9378200
	Caucasian	rs10801047, rs11175593, rs11195128, rs11805303, rs17234657, rs1893217, rs2076756, rs224136, rs3828309, rs6856616, rs7714584, rs7807268, rs9292777, rs9348876
Kidney stones (102-105)	Chinese	rs1000597, rs1256328, rs4142110, rs4293393
	Caucasian	rs1000597, rs1256328, rs219781, rs4293393
Primary biliary cholangitis (106-109)	Chinese	rs10931468, rs12134279, rs1800693, rs4979462, rs538147, rs6441286, rs7574865, rs7774434, rs8017161, rs9303277
	Caucasian	rs10931468, rs12134279, rs1800693, rs538147, rs6441286, rs7774434, rs8017161, rs9303277
Ulcerative colitis (110-117)	Chinese	rs10800309, rs11190140, rs16940202, rs17085007, rs1801274, rs2108225, rs2201841, rs2395185, rs2816958, rs3024505, rs3197999, rs4654903, rs9263739, rs9268877, rs9271366
	Caucasian	rs10800309, rs11190140, rs16940202, rs17085007, rs1801274, rs2201841, rs2816958, rs3024505, rs3197999, rs9268853, rs9268877
Heart, vascular and metabolic diseases:		
Abdominal aortic aneurysm (118-121)	Chinese	rs1333040, rs2228145, rs2383207, rs599839, rs6511720
	Caucasian	rs1333040, rs2228145, rs2383207, rs599839, rs6511720
Cerebral aneurysms (122,123)	Chinese	rs10958409, rs11661542, rs1333040, rs9298506
	Caucasian	rs10958409, rs11661542, rs1333040, rs9298506
Coronary heart disease (124-132)	Chinese	rs10757274, rs11752643, rs1333042, rs2229238, rs3782889, rs3869109, rs9349379, rs9818870
	Caucasian	rs10757274, rs2229238, rs2943634, rs3869109, rs9349379, rs9818870
Hypercholesterolemia (133,134)	Chinese	rs11576175, rs2304240, rs387865, rs4804149, rs4804636
	Caucasian	rs11576175, rs2304240, rs387865, rs4804149, rs4804636
Hypertension (93,135-137)	Chinese	rs13097326, rs13143871, rs1887320, rs2021783, rs2398162, rs35444, rs3755351, rs880315, rs9266359
	Caucasian	rs13143871, rs1887320, rs2021783, rs2398162, rs35444, rs3755351, rs5186, rs880315, rs9266359
Hypertriglyceridemia (138)	Chinese	rs1260326
	Caucasian	rs1260326
Myocardial infraction (139-143)	Chinese	rs11206510, rs17465637, rs3807989, rs4977574, rs646776, rs687289
	Caucasian	rs11206510, rs17465637, rs3184504, rs4977574, rs646776, rs687289

Obesity (144-151)	Chinese	rs11152213, rs11208659, rs13130484, rs1421085, rs1514175, rs2206277, rs3101336, rs6265, rs633715, rs6499640, rs7138803, rs925946, rs9299, rs9568856, rs987237
	Caucasian	rs11152213, rs11208659, rs13130484, rs1421085, rs1514175, rs2206277, rs3101336, rs6265, rs633715, rs6499640, rs7138803, rs925946, rs9299, rs9568856
Stroke (152-155)	Chinese	rs12530920, rs12703165, rs16851055, rs173686, rs225132, rs556621, rs7193343, rs7937106, rs879324
	Caucasian	rs12703165, rs16851055, rs173686, rs2107595, rs225132, rs556621, rs7193343, rs7937106, rs879324
Type 2 diabetes (156-176)	Chinese	rs10906115, rs11257655, rs13266634, rs1408888, rs163184, rs2074314, rs2206734, rs2237892, rs234864, rs2466293, rs391300, rs4402960, rs4430796, rs5015480, rs7756992, rs7903146, rs791595, rs7923837, rs8050136, rs9472138
	Caucasian	rs10401969, rs13266634, rs1470579, rs2237895, rs2943641, rs4430796, rs4712523, rs5015480, rs5219, rs7756992, rs7903146, rs7923837, rs864745, rs9936385
		Neurological and psychiatric diseases:
Alzheimer's disease (177)	Chinese	rs2075650
	Caucasian	rs2075650
Bipolar disorder (178,179)	Chinese	rs1064395, rs10994336
	Caucasian	rs1064395, rs10994336
Narcolepsy (180,181)	Chinese	rs2305795, rs2858884
	Caucasian	rs2305795, rs2858884
Parkinson's disease (182-189)	Chinese	rs11012, rs1994090, rs2275336, rs2395163, rs3129882, rs4538475, rs4698412, rs6532194, rs6532197, rs823128, rs823156
	Caucasian	rs11012, rs2275336, rs2395163, rs3129882, rs4538475, rs4698412, rs6532194, rs6532197, rs823128, rs823156
Schizophrenia (190-194)	Chinese	rs10489202, rs11038167, rs1635, rs16887244
	Caucasian	rs10503253, rs114002140, rs12966547, rs17504622, rs2021722, rs6932590, rs9960767
		Hypersensitivities and autoimmune diseases:
Asthma (140,195-203)	Chinese	rs11123911, rs1295686, rs1420101, rs1588265, rs17294280, rs1837253, rs1978331, rs2073643, rs2155219, rs2244012, rs2284033, rs2305480, rs2791189, rs3771166, rs4833095, rs744910, rs7521681, rs7922491, rs9268516
	Caucasian	rs1295686, rs13035227, rs1420101, rs1588265, rs17294280, rs1837253, rs1978331, rs2073643, rs2155219, rs2244012, rs2284033, rs2305480, rs2791189, rs3771166, rs4833095, rs744910, rs7521681, rs7922491, rs9268516
Psoriasis (204-214)	Chinese	rs12191877, rs1265159, rs3213094, rs3747517, rs4795067, rs7709212, rs8016947, rs999556
	Caucasian	rs11209026, rs12191877, rs12445568, rs12586317, rs20541, rs2395029, rs240993, rs2546890, rs27432, rs3213094, rs3747517, rs465969, rs4795067, rs495337, rs582757, rs610604, rs702873, rs7709212, rs8016947
Rheumatoid arthritis (93,215-220)	Chinese	rs13192471, rs1854853, rs2841277, rs6457617, rs71508903, rs9268839
	Caucasian	rs6457617, rs10488631, rs2476601, rs3087243, rs4810485, rs5029937, rs6715284, rs6920220, rs71508903, rs7574865, rs9268839, rs9826828
Systemic lupus erythematosus (221-232)	Chinese	rs10036748, rs1167796, rs12599402, rs131654, rs13277113, rs13385731, rs1913517, rs2230926, rs34330, rs3821236, rs4622329, rs4728142, rs4917014, rs4948496, rs6804441, rs7574865, rs9270650, rs9888739
	Caucasian	rs10498070, rs10516487, rs10798269, rs11574637, rs12537284, rs13239597, rs2205960, rs2736340, rs3131379, rs3821236, rs4728142, rs4963128, rs5029939, rs6445975, rs7574865, rs9888739

	Others:	
Age related macular degeneration (233-236)	Chinese	rs10490924, rs13081855, rs1713985, rs2071277
	Caucasian	rs10490924, rs13081855, rs2071277
Glaucoma (237-240)	Chinese	rs1015213, rs2487032, rs4656461
	Caucasian	rs1015213, rs4656461
Migraine (241)	Chinese	rs6478241, rs9349379
	Caucasian	rs6478241, rs9349379

Supplementary Table S1b. Diseases in which the population genetic risks of Macau were studied. SNP markers for genetic risk calculation are shown

**Diseases**	**SNP markers**
Cervical cancer (242)	rs4282438, rs8067378, rs9277952
Esophageal cancer (243-248)	rs10052657, rs10419226, rs11187842, rs11789015, rs2274223, rs2687201, rs3805322, rs398652, rs4822983, rs621559, rs738722, rs9288318
Gallstone (249)	rs3758650
Liver cancer in hepatitis B carriers (250,251)	rs455804, rs7574865, rs9272105, rs9275319
Lung cancer in never smoked women (252,253)	rs2395185, rs2736100, rs7216064, rs763317
Nasopharyngeal cancer (254-256)	rs2860580, rs29232, rs3129055, rs4411364, rs6774494, rs9260734
Polycystic ovary syndrome (257,258)	rs12478601, rs2059807, rs2268361
Sjögren's syndrome (259)	rs10168266, rs4282438, rs5029939, rs9271588
Stomach cancer (54,243,260-265)	rs10074991, rs11187842, rs1136410, rs1801133, rs2494938, rs2976392, rs4072037, rs763780, rs799917

Supplementary Table S1c. SNP markers used for genetic risk calculation of the traits associated with influenza infection

**Diseases**	**SNP markers**
Avian influenza (H7N9) susceptibility (266,267)	rs13057866, rs2070788
Influenza A (H1N1pdm09) severity of illness (267,268) (Personal communication with Prof K.Y. Yuen, manuscript submitted)	rs1130866, rs2070788, rs6487131

Supplementary Table S2. Area under curve (AUC) of genetic risk categorisation of each disease as computed by REGENT. Diseases with AUC greater than 0.6 are shown.

**Diseases**	**AUC**
Age-related macular degeneration	0.713
Alzheimer's disease	0.602
Asthma	0.664
Bladder cancer	0.614
Breast cancer	0.621
Colorectal cancer	0.607
Coronary heart disease	0.620
Crohn's disease	0.763
Endometrial cancer	0.645
Esophagael cancer	0.643
Glioma	0.617
Hypercholesterolemia	0.756
Hypertension	0.613
Hypertriglyceridemia	0.610
Kidney cancer	0.639
Lung cancer	0.609
Lung cancer in never smoked women	0.616
Myocardial infraction	0.605
Nasopharyngeal cancer	0.687
Non-Hodgkin lymphoma	0.658
Obesity	0.618
Ovarian cancer	0.644
Pancreatic cancer	0.629
Parkinson’s disease	0.635
Primary biliary cholangitis	0.696
Prostate cancer	0.671
Psoriasis	0.819
Rheumatoid arthritis	0.722
Sjögren’s syndrome	0.662
Stomach cancer	0.663
Stroke	0.601
Systemic lupus erythematosus	0.733
Thyroid cancer	0.630
Type 2 diabetes	0.691
Ulcerative colitis	0.767

Supplementary Table S3. Reference lists of the prevalence, heritability and lifetime risks of the studied diseases.

Supplementary Table S4a. Reference list of the prevalence and heritability

Disease	Prevalence	Heritability
Age-related macular degeneration	(269)	(236,270,271)
Alzheimer's disease	(272)	(273)
Asthma	(274,275)	(276-278)
Bipolar disorder	(279)	(280-282)
Bladder cancer	(283)	(284)
Breast cancer	(283)	(285)
Cerebral aneurysms	(286,287)	(288)
Cervical cancer	(283)	(289)
Colorectal cancer	(283)	(284)
Coronary heart diseases	(290-292)	(293)
Crohn's disease	(294,295)	(296)
Endometrial cancer	(283)	(297)
Esophagael cancer	(283)	(298)
Gallstone	(299)	(300,301)
Glaucoma	(302)	(303)
Glioma	(304,305)	(306)
Hypercholesterolemia	(307,308)	(309)
Hypertension	(310,311)	(312,313)
Hypertriglyceridemia	(308,314)	(315)
Kidney cancer	(283)	(297)
Kidney stones	(316-318)	(319)
Liver cancer in hepatitis B carriers	(283)	(320)
Lung cancer	(283)	(321-323)
Lung cancer in never smoked women	Not available	(324)
Migraine	(325-327)	(328)
Myocardial Infraction	(329-331)	(293)
Narcolepsy	(332,333)	Not available
Nasopharyngeal cancer	(283)	(334)
Non-Hodgkin lymphoma	(283)	(335)
Obesity	(336)	(337)
Ovarian cancer	(283)	(338)
Pancreatic cancer	(283)	(298)
Parkinson’s disease	(339,340)	(186)
Polycystic ovary syndrome	(341)	(342)
Primary biliary cholangitis	(343,344)	(345)
Prostate cancer	(283)	(346)
Psoriasis	(347-349)	(350)
Rheumatoid arthritis	(351,352)	(353)
Schizophrenia	(354,355)	(282,356)
Sjögren’s syndrome	(357)	(358)
Stomach cancer	(283)	(297)
Stroke	(359,360)	(361)
Systemic lupus erythematosus	(362,363)	(364)
Thyroid cancer	(283)	(322)
Type 2 diabetes	(365,366)	(367)
Ulcerative colitis	(368,369)	(370)

Supplementary Table S3b. Reference list of the lifetime risks

Disease	Lifetime risks
Age-related macular degeneration	(371)
Alzheimer's disease	(372)
Asthma	(373)
Bladder cancer	(374)
Breast cancer	(375)
Colorectal cancer	(375)
Coronary heart diseases	(376)
Crohn's disease	(377)
Endometrial cancer	(375)
Esophagael cancer	(374)
Glioma	(378)
Hypercholesterolemia	(379)
Hypertension	(380)
Kidney cancer	(381)
Lung cancer	(375)
Myocardial infraction	(382)
Nasopharyngeal cancer	(375)
Non-Hodgkin lymphoma	(383)
Pancreatic cancer	(374)
Parkinson’s disease	(384)
Prostate cancer	(375)
Psoriasis	(385)
Rheumatoid arthritis	(386)
Sjögren’s syndrome	(386)
Stomach cancer	(375)
Stroke	(387)
Systemic lupus erythematosus	(386)
Thyroid cancer	(375)
Type 2 diabetes	(388)

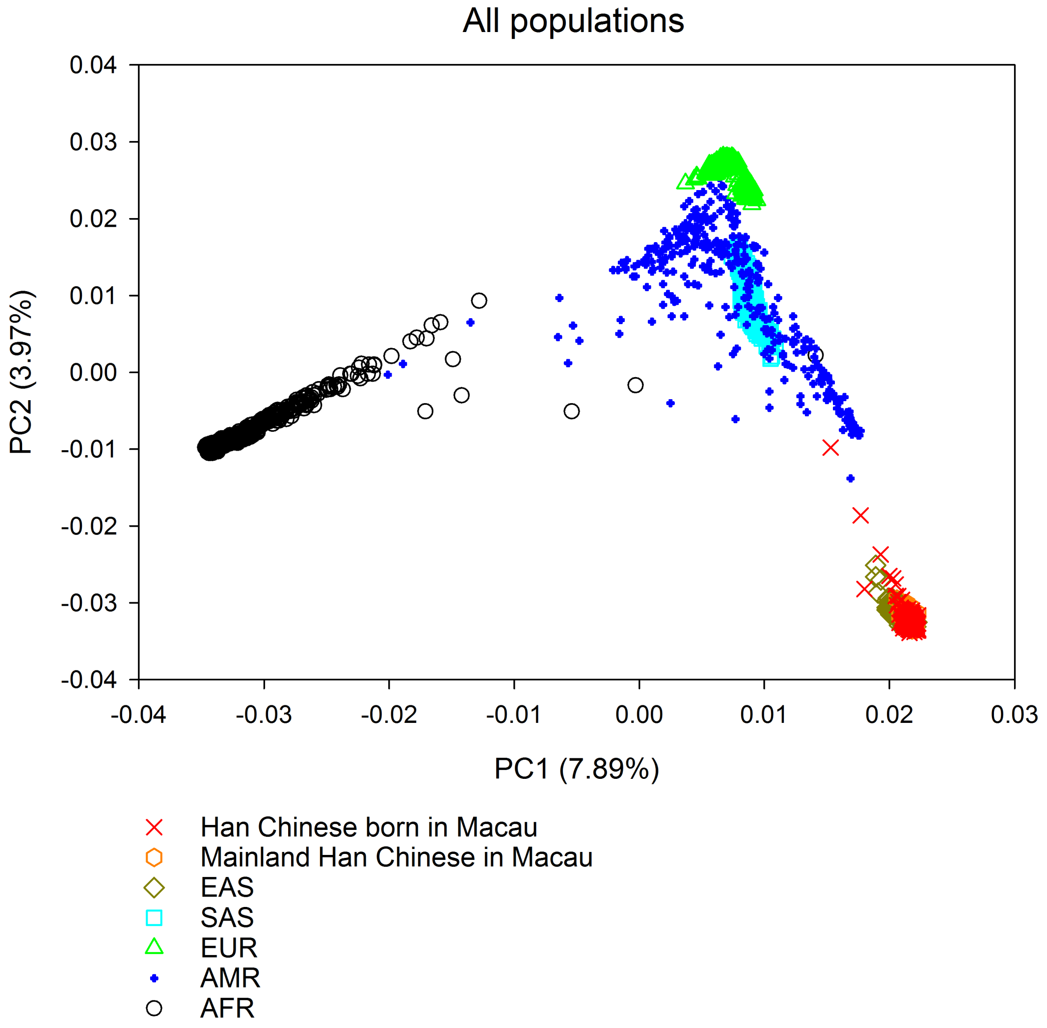

Supplementary Fig. S1 Comparison of population genetic structures among Macau and other super-populations in 1000 Genomes Project.
EAS, East Asian; SAS, South Asian; EUR, European; AMR, Ad mixed American, AFR, African.

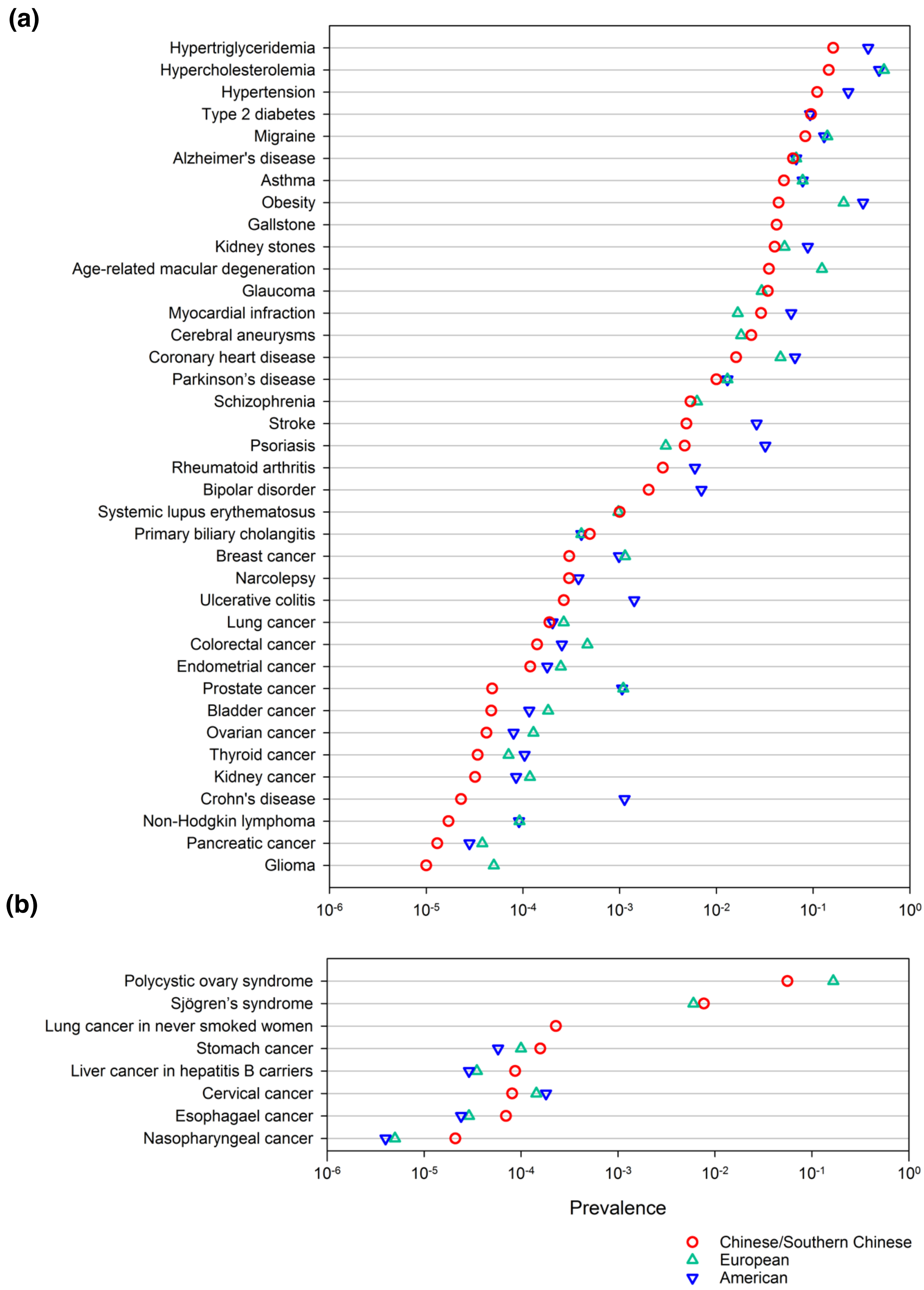

Supplementary Fig. S2 Prevalence of diseases in Chinese, European and/or American
(a) Diseases for genetic risk comparison among Macau, mainland Chinese, EUR and AMR. (b) Diseases studied in Macau only. For some of the diseases, the prevalence of European or American were unavailable.

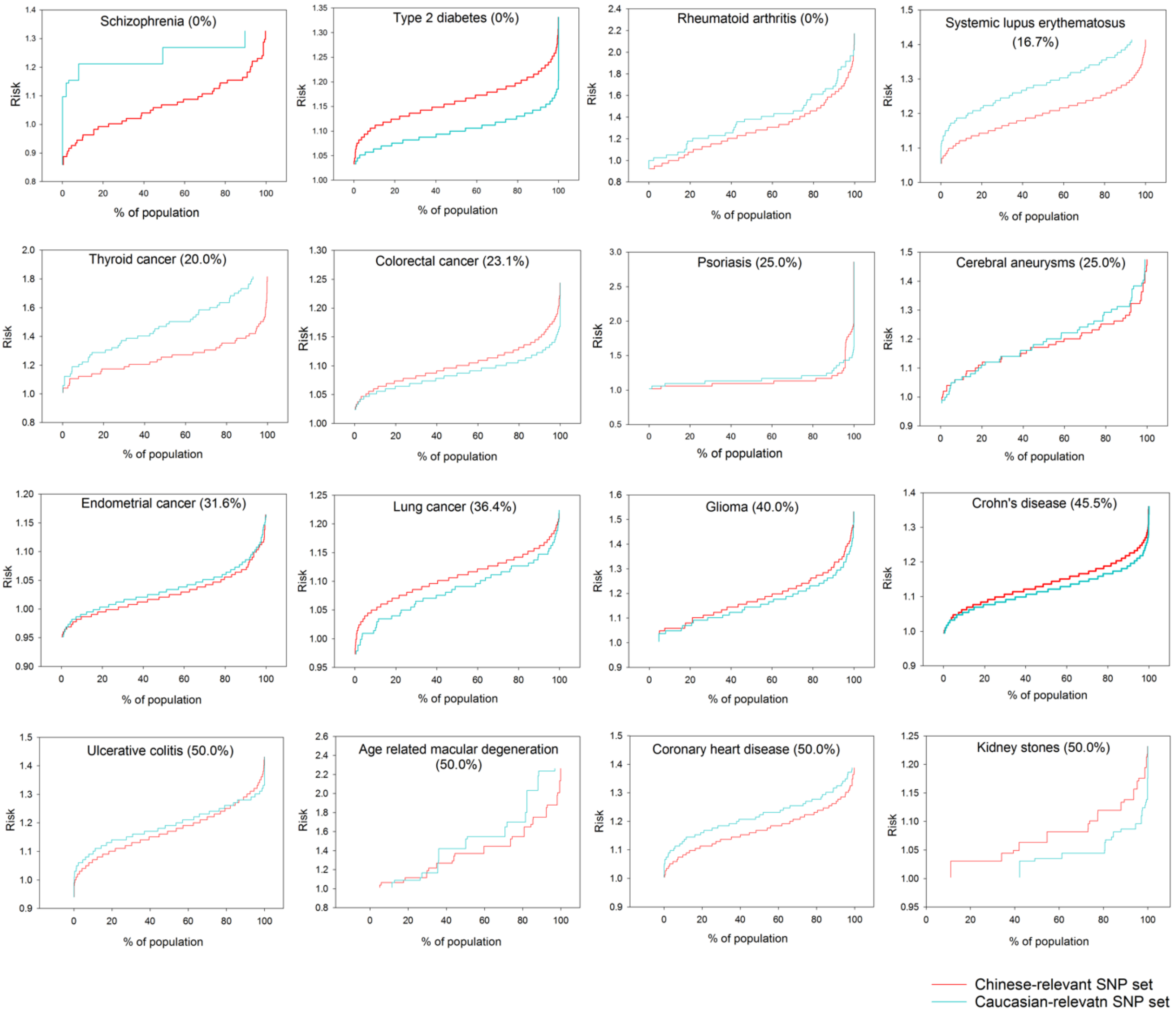

Supplementary Fig. S3 Comparison of genetic risks as calculated using Chinese-relevant SNP panels and Caucasian-relevant SNP panels on same genotype dataset of Macau population. Sixteen diseases in which the Chinese-relevant SNP panels shared ≤50.0% of similarity with the Caucasian-relevant SNP panels are shown. The similarity of each disease is given in bracket, and was calculated by:

(number of SNPs shared by Chinese and Caucasian SNP panels with same OR values) / (total number of Chinese-relevant SNPs) x 100%

When applying Caucasian-relevant SNPs to the genotypes of Macau samples, the population risks of 6 diseases were lower while 10 diseases were higher than those using Chinese-relevant SNPs (Wilcoxon sign rank tests, *P* ≤ 0.001 for all of the diseases). No significant trend of under- or over-estimation of genetic risks among the 16 analysed diseases was found when Caucasian-relevant SNPs were used for Macau population analysis (*P* = 1.000, Fisher Exact test).

(a) Diseases compared among Macau, mainland Chinese, EUR and AMR

Cancers:

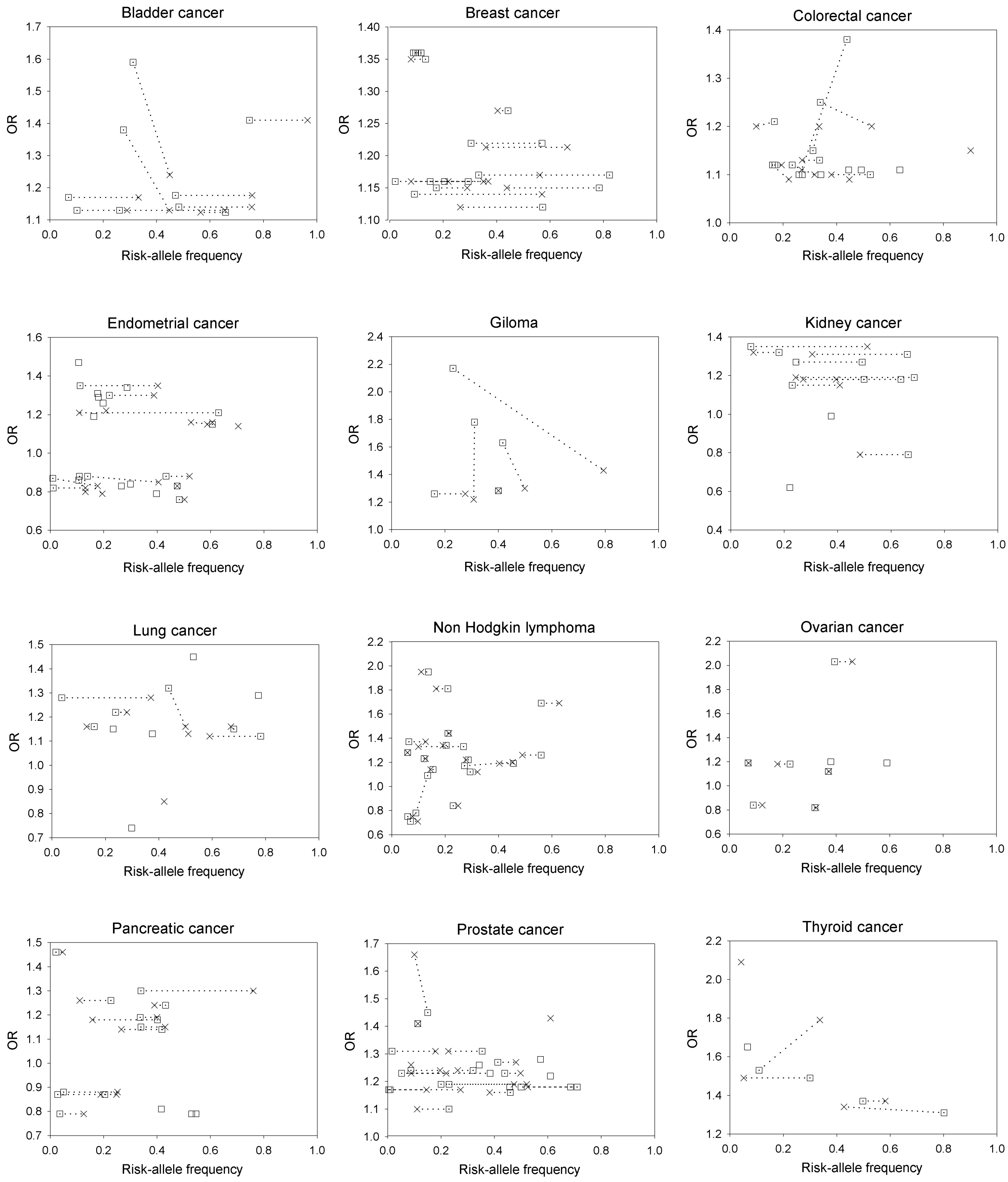

Gastrointestinal and renal diseases:

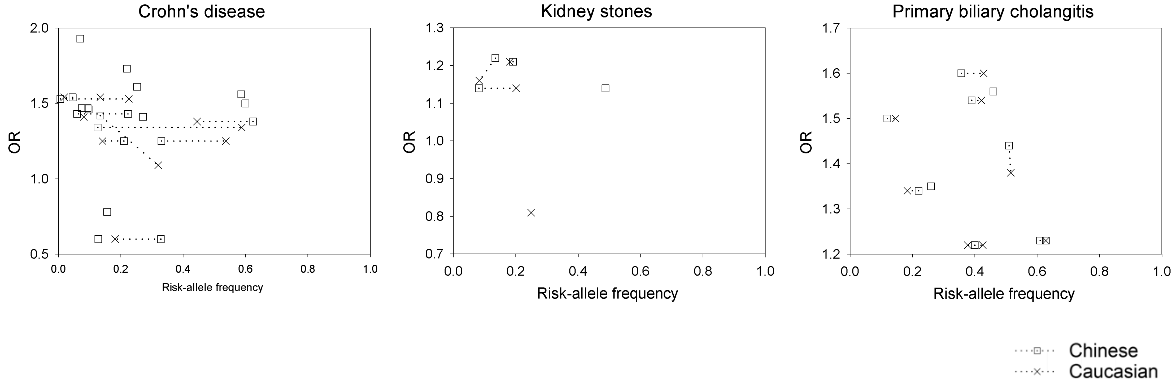

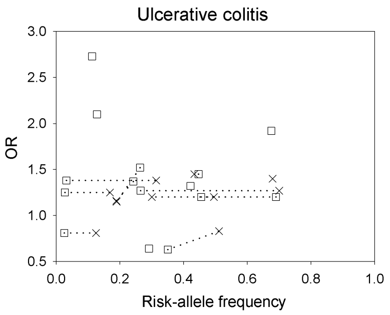

Heart, vascular and metabolic diseases:

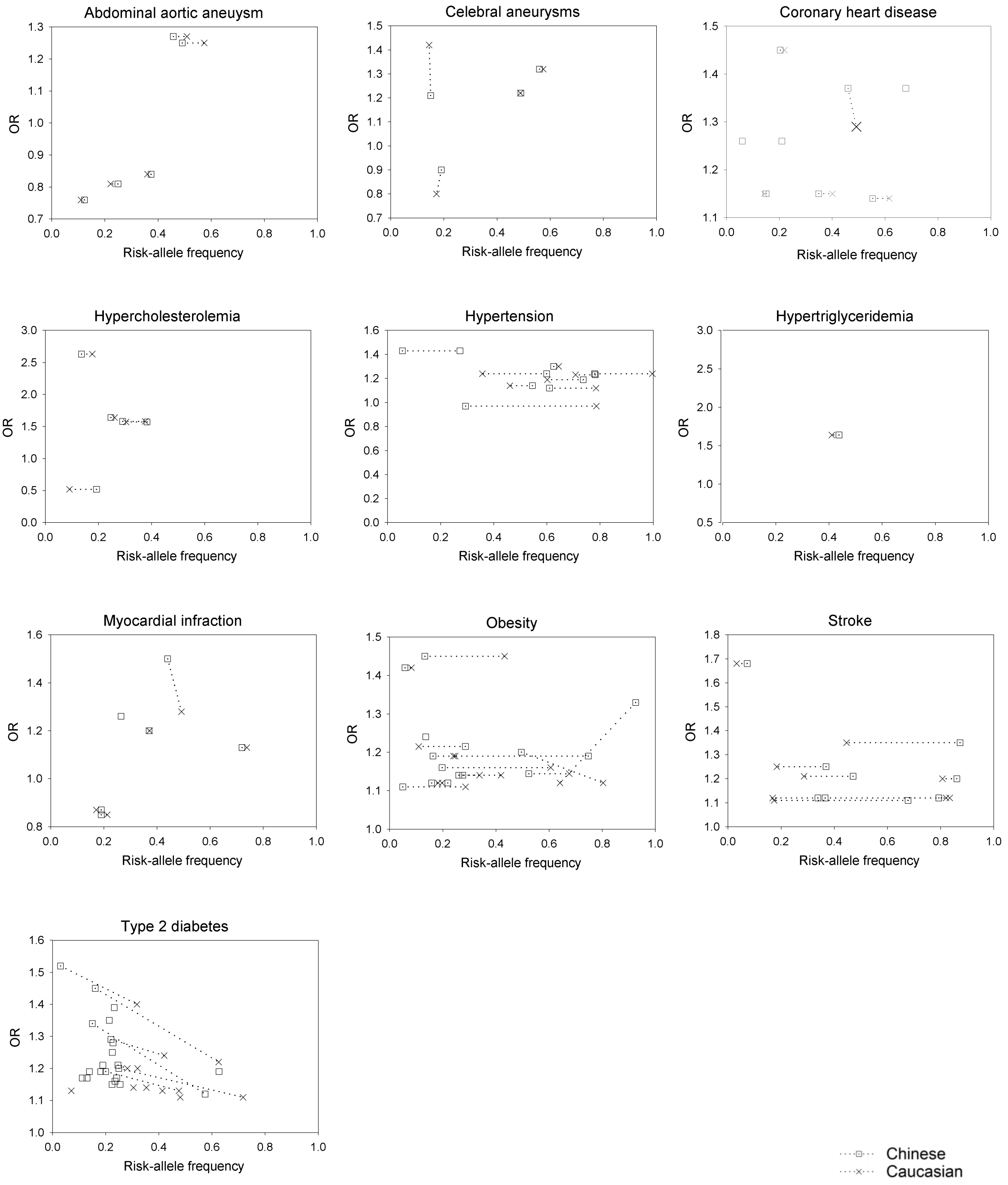

Neurological and psychiatric diseases:

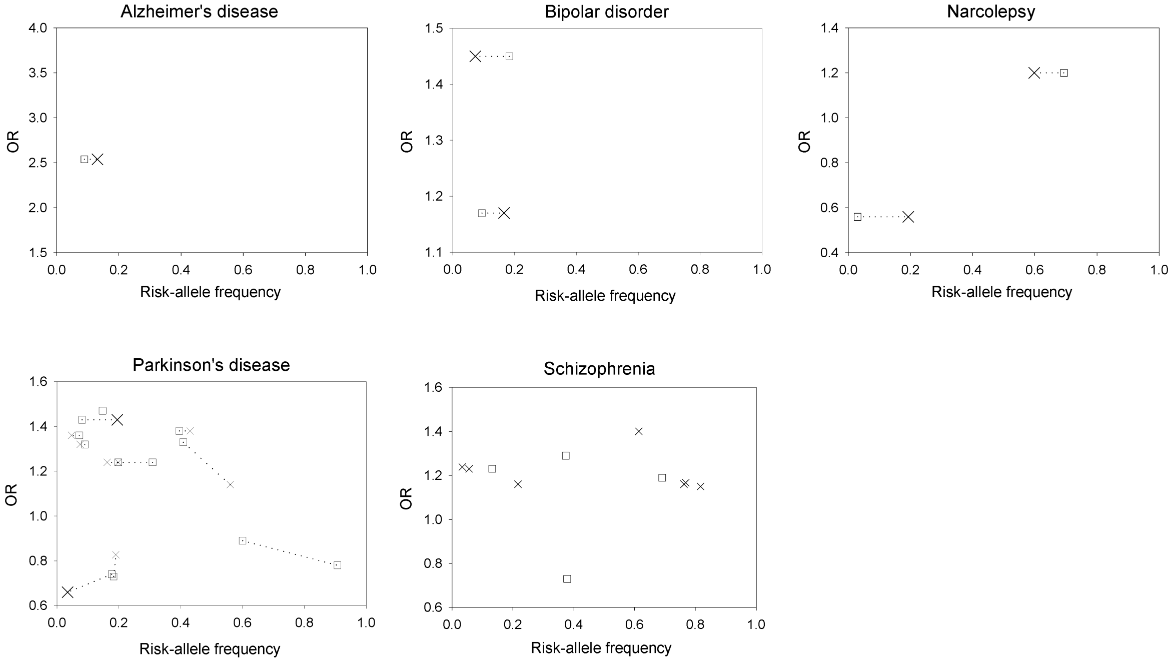

Neurological and psychiatric diseases:

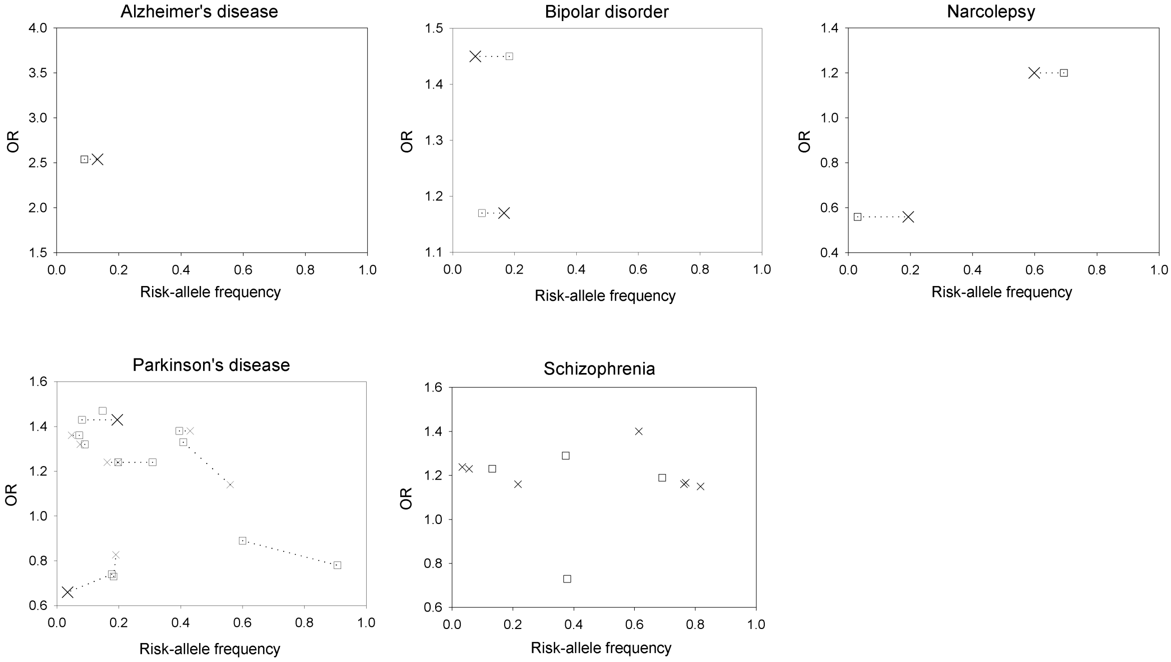

Others:

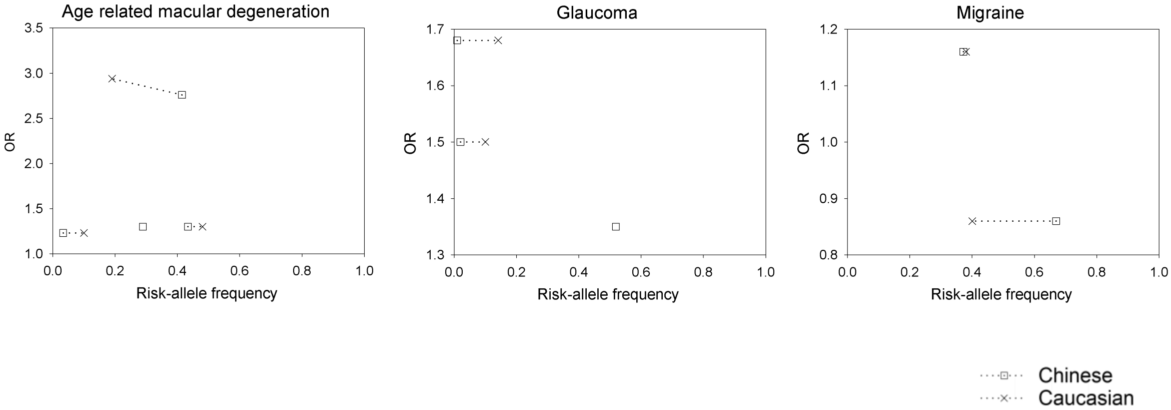

(b) Diseases for Macau population analysis:

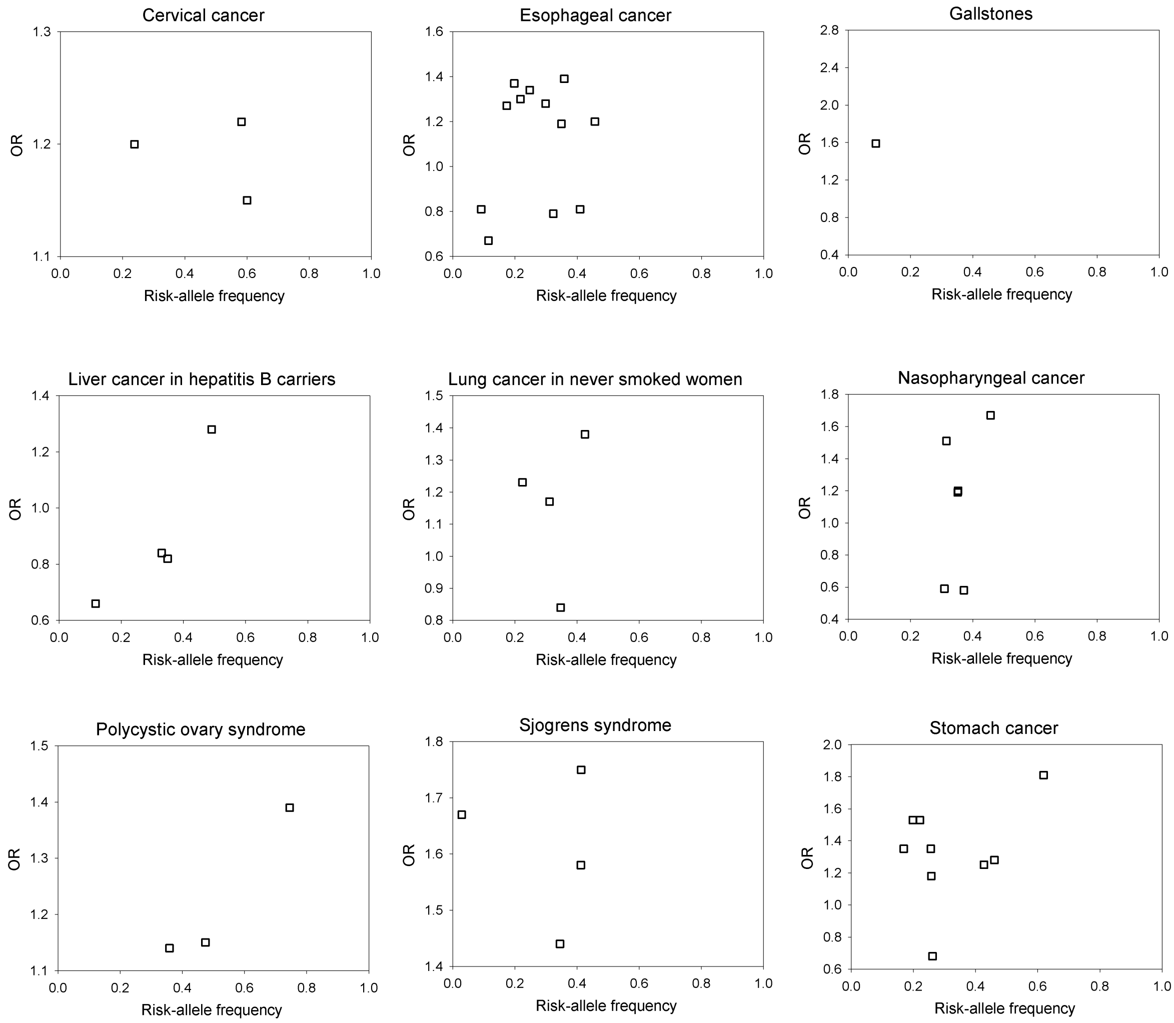

(c) Traits associated with influenza infection

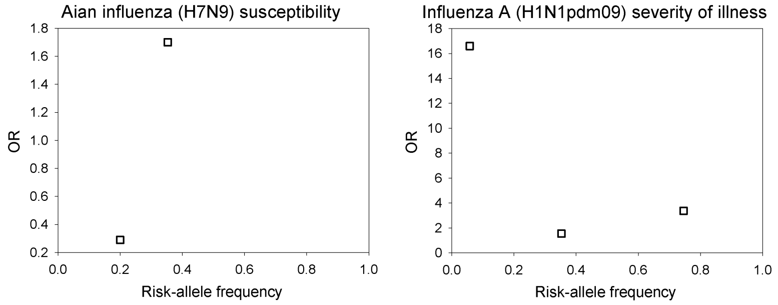

Supplementary Fig. S4 Odds ratios (ORs) and risk-allele frequencies of SNP markers used for genetic risk calculation and categorisation.
(a) SNP markers used for comparing the genetic risks among Macau, mainland Chinese, EUR and AMR. For most of the SNPs, the ORs and/or risk-allele frequencies were different between Chinese (Macau and mainland Chinese) and Caucasian (EUR and AMR). For each of these SNPs, the paired data points of Chinese (☐) and Caucasians (✕) were linked by a dotted line. For simplicity, the risk-allele frequencies of Macau were shown only for the plots of Chinese, whereas the risk-allele frequencies of EUR were shown for the plots of Caucasian. Some of the SNP markers were useful for either Chinese or Caucasian only. They were represented by individual data points without dotted line. (b) SNP markers used for calculating the genetic risk of Macau populations. (c) SNP markers

used for calculating the genetic risks of traits associated with influenza viral infection. Only ORs of Chinese were available.

References:

1 Wang, M. *et al.* Common genetic variants on 8q24 contribute to susceptibility to bladder cancer in a Chinese population. *Carcinogenesis* **30**, 991-996, (2009).

2 Wang, S., Tang, J., Wang, M., Yuan, L. & Zhang, Z. Genetic variation in PSCA and bladder cancer susceptibility in a Chinese population. *Carcinogenesis* **31**, 621-624, (2010).

3 Rothman, N. *et al.* A multi-stage genome-wide association study of bladder cancer identifies multiple susceptibility loci. *Nat Genet* **42**, 978-984, (2010).

4 Rafnar, T. *et al.* European genome-wide association study identifies SLC14A1 as a new urinary bladder cancer susceptibility gene. *Hum Mol Genet* **20**, 4268-4281, (2011).

5 Figueroa, J. D. *et al.* Genome-wide association study identifies multiple loci associated with bladder cancer risk. *Hum Mol Genet* **23**, 1387-1398, (2014).

6 Matsuda, K. *et al.* Genome-wide association study identified SNP on 15q24 associated with bladder cancer risk in Japanese population. *Hum Mol Genet* **24**, 1177-1184, (2015).

7 Easton, D. F. *et al.* Genome-wide association study identifies novel breast cancer susceptibility loci. *Nature* **447**, 1087-1093, (2007).

8 Thomas, G. *et al.* A multistage genome-wide association study in breast cancer identifies two new risk alleles at 1p11.2 and 14q24.1 (RAD51L1). *Nat Genet* **41**, 579-584, (2009).

9 Turnbull, C. *et al.* Genome-wide association study identifies five new breast cancer susceptibility loci. *Nat Genet* **42**, 504-507, (2010).

10 Fletcher, O. *et al.* Novel breast cancer susceptibility locus at 9q31.2: results of a genome-wide association study. *J Natl Cancer Inst* **103**, 425-435, (2011).

11 Siddiq, A. *et al.* A meta-analysis of genome-wide association studies of breast cancer identifies two novel susceptibility loci at 6q14 and 20q11. *Hum Mol Genet* **21**, 5373-5384, (2012).

12 Garcia-Closas, M. *et al.* Genome-wide association studies identify four ER negative-specific breast cancer risk loci. *Nat Genet* **45**, 392-398, 398e391-392, (2013).

13 Michailidou, K. *et al.* Large-scale genotyping identifies 41 new loci associated with breast cancer risk. *Nat Genet* **45**, 353-361, 361e351-352, (2013).

14 Low, S. K. *et al.* Genome-wide association study of breast cancer in the Japanese population. *PLoS One* **8**, e76463, (2013).

15 Ahsan, H. *et al.* A genome-wide association study of early-onset breast cancer identifies PFKM as a novel breast cancer gene and supports a common genetic spectrum for breast cancer at any age. *Cancer Epidemiol Biomarkers Prev* **23**, 658-669, (2014).

16 Purrington, K. S. *et al.* Genome-wide association study identifies 25 known breast cancer susceptibility loci as risk factors for triple-negative breast cancer. *Carcinogenesis* **35**, 1012-1019, (2014).

17 Broderick, P. *et al.* A genome-wide association study shows that common alleles of SMAD7 influence colorectal cancer risk. *Nat Genet* **39**, 1315-1317, (2007).

18 Study, C. *et al.* Meta-analysis of genome-wide association data identifies four new susceptibility loci for colorectal cancer. *Nat Genet* **40**, 1426-1435, (2008).

19 Tenesa, A. *et al.* Genome-wide association scan identifies a colorectal cancer susceptibility locus on 11q23 and replicates risk loci at 8q24 and 18q21. *Nat Genet* **40**, 631-637, (2008).

20 Slattery, M. L. *et al.* Increased risk of colon cancer associated with a genetic polymorphism of SMAD7. *Cancer Res* **70**, 1479-1485, (2010).

21 Xiong, F. *et al.* Risk of genome-wide association study-identified genetic variants for colorectal cancer in a Chinese population. *Cancer Epidemiol Biomarkers Prev* **19**, 1855-1861, (2010).

22 Spain, S. L. *et al.* Refinement of the associations between risk of colorectal cancer and polymorphisms on chromosomes 1q41 and 12q13.13. *Hum Mol Genet* **21**, 934-946, (2012).

23 Peters, U. *et al.* Identification of Genetic Susceptibility Loci for Colorectal Tumors in a Genome-Wide Meta-analysis. *Gastroenterology* **144**, 799-807 e724, (2013).

24 Zhang, B. *et al.* Genome-wide association study identifies a new SMAD7 risk variant associated with colorectal cancer risk in East Asians. *Int J Cancer* **135**, 948-955, (2014).

25 Whiffin, N. *et al.* Identification of susceptibility loci for colorectal cancer in a genome-wide meta-analysis. *Hum Mol Genet* **23**, 4729-4737, (2014).

26 Zhang, B. *et al.* Large-scale genetic study in East Asians identifies six new loci associated with colorectal cancer risk. *Nat Genet* **46**, 533-542, (2014).

27 Delahanty, R. J. *et al.* Association of obesity-related genetic variants with endometrial cancer risk: a report from the Shanghai Endometrial Cancer Genetics Study. *Am J Epidemiol* **174**, 1115-1126, (2011).

28 Spurdle, A. B. *et al.* Genome-wide association study identifies a common variant associated with risk of endometrial cancer. *Nat Genet* **43**, 451-454, (2011).

29 Long, J. *et al.* Genome-wide association study identifies a possible susceptibility locus for endometrial cancer. *Cancer Epidemiol Biomarkers Prev* **21**, 980-987, (2012).

30 Setiawan, V. W. *et al.* HNF1B and endometrial cancer risk: results from the PAGE study. *PLoS One* **7**, e30390, (2012).

31 Teng, Y., He, C., Zuo, X. & Li, X. Catechol-O-methyltransferase and cytochrome P-450 1B1 polymorphisms and endometrial cancer risk: a meta-analysis. *Int J Gynecol Cancer* **23**, 422-430, (2013).

32 Gu, F. *et al.* Common genetic variants in the 9p21 region and their associations with multiple tumours. *Br J Cancer* **108**, 1378-1386, (2013).

33 De Vivo, I. *et al.* Genome-wide association study of endometrial cancer in E2C2. *Hum Genet* **133**, 211-224, (2014).

34 Shete, S. *et al.* Genome-wide association study identifies five susceptibility loci for glioma. *Nat Genet* **41**, 899-904, (2009).

35 Sanson, M. *et al.* Chromosome 7p11.2 (EGFR) variation influences glioma risk. *Hum Mol Genet* **20**, 2897-2904, (2011).

36 Chen, H. *et al.* Association of sequence variants on chromosomes 20, 11, and 5 (20q13.33, 11q23.3, and 5p15.33) with glioma susceptibility in a Chinese population. *Am J Epidemiol* **173**, 915-922, (2011).

37 Rajaraman, P. *et al.* Genome-wide association study of glioma and meta-analysis. *Hum Genet* **131**, 1877-1888, (2012).

38 Moore, L. E. *et al.* Apolipoprotein E/C1 locus variants modify renal cell carcinoma risk. *Cancer Res* **69**, 8001-8008, (2009).

39 Andreotti, G. *et al.* Variants in blood pressure genes and the risk of renal cell carcinoma. *Carcinogenesis* **31**, 614-620, (2010).

40 Cao, Q. *et al.* Genetic polymorphisms in APE1 are associated with renal cell carcinoma risk in a Chinese population. *Mol Carcinog* **50**, 863-870, (2011).

41 Wu, X. *et al.* A genome-wide association study identifies a novel susceptibility locus for renal cell carcinoma on 12p11.23. *Hum Mol Genet* **21**, 456-462, (2012).

42 Li, W. Q. *et al.* Genetic polymorphisms in the 9p21 region associated with risk of multiple cancers. *Carcinogenesis* **35**, 2698-2705, (2014).

43 Lv, C., Bai, Z., Liu, Z., Luo, P. & Zhang, J. Renal cell carcinoma risk is associated with the interactions of APOE, VHL and MTHFR gene polymorphisms. *Int J Clin Exp Pathol* **8**, 5781-5786, (2015).

44 Zhao, R. *et al.* Genetic Variants in Caveolin-1 and RhoA/ROCK1 Are Associated with Clear Cell Renal Cell Carcinoma Risk in a Chinese Population. *PLoS One* **10**, e0128771, (2015).

45 Hu, Z. *et al.* Functional polymorphisms of matrix metalloproteinase-9 are associated with risk of occurrence and metastasis of lung cancer. *Clin Cancer Res* **11**, 5433-5439, (2005).

46 McKay, J. D. *et al.* Lung cancer susceptibility locus at 5p15.33. *Nat Genet* **40**, 1404-1406, (2008).

47 Amos, C. I. *et al.* Genome-wide association scan of tag SNPs identifies a susceptibility locus for lung cancer at 15q25.1. *Nat Genet* **40**, 616-622, (2008).

48 Landi, M. T. *et al.* A genome-wide association study of lung cancer identifies a region of chromosome 5p15 associated with risk for adenocarcinoma. *Am J Hum Genet* **85**, 679-691, (2009).

49 Broderick, P. *et al.* Deciphering the impact of common genetic variation on lung cancer risk: a genome-wide association study. *Cancer Res* **69**, 6633-6641, (2009).

50 Wu, C. *et al.* Genetic variants on chromosome 15q25 associated with lung cancer risk in Chinese populations. *Cancer Res* **69**, 5065-5072, (2009).

51 Truong, T. *et al.* Replication of lung cancer susceptibility loci at chromosomes 15q25, 5p15, and 6p21: a pooled analysis from the International Lung Cancer Consortium. *J Natl Cancer Inst* **102**, 959-971, (2010).

52 Hu, Z. *et al.* A genome-wide association study identifies two new lung cancer susceptibility loci at 13q12.12 and 22q12.2 in Han Chinese. *Nat Genet* **43**, 792-796, (2011).

53 Dong, J. *et al.* Association analyses identify multiple new lung cancer susceptibility loci and their interactions with smoking in the Chinese population. *Nat Genet* **44**, 895-899, (2012).

54 Jin, G. *et al.* Genetic variants at 6p21.1 and 7p15.3 are associated with risk of multiple cancers in Han Chinese. *Am J Hum Genet* **91**, 928-934, (2012).

55 Dong, J. *et al.* Genome-wide association study identifies a novel susceptibility locus at 12q23.1 for lung squamous cell carcinoma in han chinese. *PLoS Genet* **9**, e1003190, (2013).

56 Wang, Y. *et al.* Rare variants of large effect in BRCA2 and CHEK2 affect risk of lung cancer. *Nat Genet* **46**, 736-741, (2014).

57 Wang, S. S. *et al.* Common genetic variants in proinflammatory and other immunoregulatory genes and risk for non-Hodgkin lymphoma. *Cancer Res* **66**, 9771-9780, (2006).

58 Skibola, C. F. *et al.* Genetic variants at 6p21.33 are associated with susceptibility to follicular lymphoma. *Nat Genet* **41**, 873-875, (2009).

59 Skibola, C. F. *et al.* Tumor necrosis factor (TNF) and lymphotoxin-alpha (LTA) polymorphisms and risk of non-Hodgkin lymphoma in the InterLymph Consortium. *Am J Epidemiol* **171**, 267-276, (2010).

60 Conde, L. *et al.* Genome-wide association study of follicular lymphoma identifies a risk locus at 6p21.32. *Nat Genet* **42**, 661-664, (2010).

61 Hosgood, H. D., 3rd *et al.* IL10 and TNF variants and risk of non-Hodgkin lymphoma among three Asian populations. *Int J Hematol* **97**, 793-799, (2013).

62 Vijai, J. *et al.* Susceptibility loci associated with specific and shared subtypes of lymphoid malignancies. *PLoS Genet* **9**, e1003220, (2013).

63 Tan, D. E. *et al.* Genome-wide association study of B cell non-Hodgkin lymphoma identifies 3q27 as a susceptibility locus in the Chinese population. *Nat Genet* **45**, 804-807, (2013).

64 Dai, Z. M. *et al.* Association of the four common polymorphisms in interleukin-10 (rs1800890, rs1800896, rs1800871, and rs1800872) with non-Hodgkin's lymphoma risk: a meta-analysis. *Int J Clin Exp Med* **7**, 4720-4733, (2014).

65 Rendleman, J. *et al.* Genetic variation in DNA repair pathways and risk of non-Hodgkin's lymphoma. *PLoS One* **9**, e101685, (2014).

66 Skibola, C. F. *et al.* Genome-wide association study identifies five susceptibility loci for follicular lymphoma outside the HLA region. *Am J Hum Genet* **95**, 462-471, (2014).

67 Quaye, L. *et al.* Association between common germline genetic variation in 94 candidate genes or regions and risks of invasive epithelial ovarian cancer. *PLoS One* **4**, e5983, (2009).

68 Goode, E. L. *et al.* A genome-wide association study identifies susceptibility loci for ovarian cancer at 2q31 and 8q24. *Nat Genet* **42**, 874-879, (2010).

69 Pharoah, P. D. *et al.* GWAS meta-analysis and replication identifies three new susceptibility loci for ovarian cancer. *Nat Genet* **45**, 362-370, 370e361-362, (2013).

70 Chen, K. *et al.* Genome-wide association study identifies new susceptibility loci for epithelial ovarian cancer in Han Chinese women. *Nat Commun* **5**, 4682, (2014).

71 Wang, Y. *et al.* Genetic variants in matrix metalloproteinase genes as disposition factors for ovarian cancer risk, survival, and clinical outcome. *Mol Carcinog* **54**, 430-439, (2015).

72 Wu, C. *et al.* Genome-wide association study identifies five loci associated with susceptibility to pancreatic cancer in Chinese populations. *Nat Genet* **44**, 62-66, (2011).

73 Li, D. *et al.* Pathway analysis of genome-wide association study data highlights pancreatic development genes as susceptibility factors for pancreatic cancer. *Carcinogenesis* **33**, 1384-1390, (2012).

74 Wolpin, B. M. *et al.* Genome-wide association study identifies multiple susceptibility loci for pancreatic cancer. *Nat Genet* **46**, 994-1000, (2014).

75 Childs, E. J. *et al.* Common variation at 2p13.3, 3q29, 7p13 and 17q25.1 associated with susceptibility to pancreatic cancer. *Nat Genet* **47**, 911-916, (2015).

76 Thomas, G. *et al.* Multiple loci identified in a genome-wide association study of prostate cancer. *Nat Genet* **40**, 310-315, (2008).

77 Gudmundsson, J. *et al.* Common sequence variants on 2p15 and Xp11.22 confer susceptibility to prostate cancer. *Nat Genet* **40**, 281-283, (2008).

78 Eeles, R. A. *et al.* Multiple newly identified loci associated with prostate cancer susceptibility. *Nat Genet* **40**, 316-321, (2008).

79 Gudmundsson, J. *et al.* Genome-wide association and replication studies identify four variants associated with prostate cancer susceptibility. *Nat Genet* **41**, 1122-1126, (2009).

80 Takata, R. *et al.* Genome-wide association study identifies five new susceptibility loci for prostate cancer in the Japanese population. *Nat Genet* **42**, 751-754, (2010).

81 Schumacher, F. R. *et al.* Genome-wide association study identifies new prostate cancer susceptibility loci. *Hum Mol Genet* **20**, 3867-3875, (2011).

82 Xu, J. *et al.* Genome-wide association study in Chinese men identifies two new prostate cancer risk loci at 9q31.2 and 19q13.4. *Nat Genet* **44**, 1231-1235, (2012).

83 Cheng, I. *et al.* Evaluating genetic risk for prostate cancer among Japanese and Latinos. *Cancer Epidemiol Biomarkers Prev* **21**, 2048-2058, (2012).

84 Lange, E. M. *et al.* Genome-wide association scan for variants associated with early-onset prostate cancer. *PLoS One* **9**, e93436, (2014).

85 Berndt, S. I. *et al.* Two susceptibility loci identified for prostate cancer aggressiveness. *Nat Commun* **6**, 6889, (2015).

86 Gudmundsson, J. *et al.* Common variants on 9q22.33 and 14q13.3 predispose to thyroid cancer in European populations. *Nat Genet* **41**, 460-464, (2009).

87 Gudmundsson, J. *et al.* Discovery of common variants associated with low TSH levels and thyroid cancer risk. *Nat Genet* **44**, 319-322, (2012).

88 Wang, Y. L. *et al.* Confirmation of papillary thyroid cancer susceptibility loci identified by genome-wide association studies of chromosomes 14q13, 9q22, 2q35 and 8p12 in a Chinese population. *J Med Genet* **50**, 689-695, (2013).

89 Kohler, A. *et al.* Genome-wide association study on differentiated thyroid cancer. *J Clin Endocrinol Metab* **98**, E1674-1681, (2013).

90 Wang, C. & Ai, Z. Association of XRCC1 polymorphisms with thyroid cancer risk. *Tumour Biol* **35**, 4791-4797, (2014).

91 Yuan, K. *et al.* Association between x-ray repair cross-complementing group 3 (XRCC3) genetic polymorphisms and papillary thyroid cancer susceptibility in a Chinese Han population. *Tumour Biol* **37**, 979-987, (2016).

92 Duerr, R. H. *et al.* A genome-wide association study identifies IL23R as an inflammatory bowel disease gene. *Science* **314**, 1461-1463, (2006).

93 Wellcome Trust Case Control, C. Genome-wide association study of 14,000 cases of seven common diseases and 3,000 shared controls. *Nature* **447**, 661-678, (2007).

94 Rioux, J. D. *et al.* Genome-wide association study identifies new susceptibility loci for Crohn disease and implicates autophagy in disease pathogenesis. *Nat Genet* **39**, 596-604, (2007).

95 Libioulle, C. *et al.* Novel Crohn disease locus identified by genome-wide association maps to a gene desert on 5p13.1 and modulates expression of PTGER4. *PLoS Genet* **3**, e58, (2007).

96 Parkes, M. *et al.* Sequence variants in the autophagy gene IRGM and multiple other replicating loci contribute to Crohn's disease susceptibility. *Nat Genet* **39**, 830-832, (2007).

97 Barrett, J. C. *et al.* Genome-wide association defines more than 30 distinct susceptibility loci for Crohn's disease. *Nat Genet* **40**, 955-962, (2008).

98 Franke, A. *et al.* Genome-wide meta-analysis increases to 71 the number of confirmed Crohn's disease susceptibility loci. *Nat Genet* **42**, 1118-1125, (2010).

99 Yamazaki, K. *et al.* A genome-wide association study identifies 2 susceptibility Loci for Crohn's disease in a Japanese population. *Gastroenterology* **144**, 781-788, (2013).

100 Julia, A. *et al.* A genome-wide association study on a southern European population identifies a new Crohn's disease susceptibility locus at RBX1-EP300. *Gut* **62**, 1440-1445, (2013).

101 Yang, S. K. *et al.* Genome-wide association study of Crohn's disease in Koreans revealed three new susceptibility loci and common attributes of genetic susceptibility across ethnic populations. *Gut* **63**, 80-87, (2014).

102 Thorleifsson, G. *et al.* Sequence variants in the CLDN14 gene associate with kidney stones and bone mineral density. *Nat Genet* **41**, 926-930, (2009).

103 Gudbjartsson, D. F. *et al.* Association of variants at UMOD with chronic kidney disease and kidney stones-role of age and comorbid diseases. *PLoS Genet* **6**, e1001039, (2010).

104 Urabe, Y. *et al.* A genome-wide association study of nephrolithiasis in the Japanese population identifies novel susceptible Loci at 5q35.3, 7p14.3, and 13q14.1. *PLoS Genet* **8**, e1002541, (2012).

105 Oddsson, A. *et al.* Common and rare variants associated with kidney stones and biochemical traits. *Nat Commun* **6**, 7975, (2015).

106 Hirschfield, G. M. *et al.* Primary biliary cirrhosis associated with HLA, IL12A, and IL12RB2 variants. *N Engl J Med* **360**, 2544-2555, (2009).

107 Liu, X. *et al.* Genome-wide meta-analyses identify three loci associated with primary biliary cirrhosis. *Nat Genet* **42**, 658-660, (2010).

108 Mells, G. F. *et al.* Genome-wide association study identifies 12 new susceptibility loci for primary biliary cirrhosis. *Nat Genet* **43**, 329-332, (2011).

109 Nakamura, M. *et al.* Genome-wide association study identifies TNFSF15 and POU2AF1 as susceptibility loci for primary biliary cirrhosis in the Japanese population. *Am J Hum Genet* **91**, 721-728, (2012).

110 Franke, A. *et al.* Sequence variants in IL10, ARPC2 and multiple other loci contribute to ulcerative colitis susceptibility. *Nat Genet* **40**, 1319-1323, (2008).

111 Silverberg, M. S. *et al.* Ulcerative colitis-risk loci on chromosomes 1p36 and 12q15 found by genome-wide association study. *Nat Genet* **41**, 216-220, (2009).

112 Asano, K. *et al.* A genome-wide association study identifies three new susceptibility loci for ulcerative colitis in the Japanese population. *Nat Genet* **41**, 1325-1329, (2009).

113 McGovern, D. P. *et al.* Genome-wide association identifies multiple ulcerative colitis susceptibility loci. *Nat Genet* **42**, 332-337, (2010).

114 Anderson, C. A. *et al.* Meta-analysis identifies 29 additional ulcerative colitis risk loci, increasing the number of confirmed associations to 47. *Nat Genet* **43**, 246-252, (2011).

115 Jostins, L. *et al.* Host-microbe interactions have shaped the genetic architecture of inflammatory bowel disease. *Nature* **491**, 119-124, (2012).

116 Yang, S. K. *et al.* Genome-wide association study of ulcerative colitis in Koreans suggests extensive overlapping of genetic susceptibility with Caucasians. *Inflamm Bowel Dis* **19**, 954-966, (2013).

117 Julia, A. *et al.* A genome-wide association study identifies a novel locus at 6q22.1 associated with ulcerative colitis. *Hum Mol Genet* **23**, 6927-6934, (2014).

118 Gretarsdottir, S. *et al.* Genome-wide association study identifies a sequence variant within the DAB2IP gene conferring susceptibility to abdominal aortic aneurysm. *Nat Genet* **42**, 692-697, (2010).

119 Harrison, S. C. *et al.* Interleukin-6 receptor pathways in abdominal aortic aneurysm. *Eur Heart J* **34**, 3707-3716, (2013).

120 Jones, G. T. *et al.* A sequence variant associated with sortilin-1 (SORT1) on 1p13.3 is independently associated with abdominal aortic aneurysm. *Hum Mol Genet* **22**, 2941-2947, (2013).

121 Bradley, D. T. *et al.* A variant in LDLR is associated with abdominal aortic aneurysm. *Circ Cardiovasc Genet* **6**, 498-504, (2013).

122 Bilguvar, K. *et al.* Susceptibility loci for intracranial aneurysm in European and Japanese populations. *Nat Genet* **40**, 1472-1477, (2008).

123 Yasuno, K. *et al.* Genome-wide association study of intracranial aneurysm identifies three new risk loci. *Nat Genet* **42**, 420-425, (2010).

124 Samani, N. J. *et al.* Genomewide association analysis of coronary artery disease. *N Engl J Med* **357**, 443-453, (2007).

125 Anderson, J. L. *et al.* Genetic variation at the 9p21 locus predicts angiographic coronary artery disease prevalence but not extent and has clinical utility. *Am Heart J* **156**, 1155-1162 e1152, (2008).

126 Erdmann, J. *et al.* New susceptibility locus for coronary artery disease on chromosome 3q22.3. *Nat Genet* **41**, 280-282, (2009).

127 Schaefer, A. S. *et al.* Identification of a shared genetic susceptibility locus for coronary heart disease and periodontitis. *PLoS Genet* **5**, e1000378, (2009).

128 Coronary Artery Disease Genetics Consortium. A genome-wide association study in Europeans and South Asians identifies five new loci for coronary artery disease. *Nat Genet* **43**, 339-344, (2011).

129 Lu, X. *et al.* Genome-wide association study in Han Chinese identifies four new susceptibility loci for coronary artery disease. *Nat Genet* **44**, 890-894, (2012).

130 Takeuchi, F. *et al.* Genome-wide association study of coronary artery disease in the Japanese. *Eur J Hum Genet* **20**, 333-340, (2012).

131 Davies, R. W. *et al.* A genome-wide association study for coronary artery disease identifies a novel susceptibility locus in the major histocompatibility complex. *Circ Cardiovasc Genet* **5**, 217-225, (2012).

132 Lee, J. Y. *et al.* A genome-wide association study of a coronary artery disease risk variant. *J Hum Genet* **58**, 120-126, (2013).

133 Ou, Z. *et al.* The genetic polymorphisms of cathepsin S were associated with metabolic disorders in a Chinese Han population. *Gene* **526**, 385-389, (2013).

134 Oosterveer, D. M. *et al.* Low-density lipoprotein receptor mutations generate synthetic genome-wide associations. *Eur J Hum Genet* **21**, 563-566, (2013).

135 Bonnardeaux, A. *et al.* Angiotensin II type 1 receptor gene polymorphisms in human essential hypertension. *Hypertension* **24**, 63-69, (1994).

136 Kato, N. *et al.* High-density association study and nomination of susceptibility genes for hypertension in the Japanese National Project. *Hum Mol Genet* **17**, 617-627, (2008).

137 Lu, X. *et al.* Genome-wide association study in Chinese identifies novel loci for blood pressure and hypertension. *Hum Mol Genet* **24**, 865-874, (2015).

138 Johansen, C. T. *et al.* An increased burden of common and rare lipid-associated risk alleles contributes to the phenotypic spectrum of hypertriglyceridemia. *Arterioscler Thromb Vasc Biol* **31**, 1916-1926, (2011).

139 Myocardial Infarction Genetics, C. *et al.* Genome-wide association of early-onset myocardial infarction with single nucleotide polymorphisms and copy number variants. *Nat Genet* **41**, 334-341, (2009).

140 Gudbjartsson, D. F. *et al.* Sequence variants affecting eosinophil numbers associate with asthma and myocardial infarction. *Nat Genet* **41**, 342-347, (2009).

141 Reilly, M. P. *et al.* Identification of ADAMTS7 as a novel locus for coronary atherosclerosis and association of ABO with myocardial infarction in the presence of coronary atherosclerosis: two genome-wide association studies. *Lancet* **377**, 383-392, (2011).

142 Wang, Y. *et al.* Genetic variants associated with myocardial infarction and the risk factors in Chinese population. *PLoS One* **9**, e86332, (2014).

143 Chen, S. *et al.* Genomic variant in CAV1 increases susceptibility to coronary artery disease and myocardial infarction. *Atherosclerosis* **246**, 148-156, (2016).

144 Thorleifsson, G. *et al.* Genome-wide association yields new sequence variants at seven loci that associate with measures of obesity. *Nat Genet* **41**, 18-24, (2009).

145 Cheung, C. Y. *et al.* Obesity susceptibility genetic variants identified from recent genome-wide association studies: implications in a chinese population. *J Clin Endocrinol Metab* **95**, 1395-1403, (2010).

146 Bradfield, J. P. *et al.* A genome-wide association meta-analysis identifies new childhood obesity loci. *Nat Genet* **44**, 526-531, (2012).

147 Berndt, S. I. *et al.* Genome-wide meta-analysis identifies 11 new loci for anthropometric traits and provides insights into genetic architecture. *Nat Genet* **45**, 501-512, (2013).

148 Wheeler, E. *et al.* Genome-wide SNP and CNV analysis identifies common and low-frequency variants associated with severe early-onset obesity. *Nat Genet* **45**, 513-517, (2013).

149 Magi, R. *et al.* Contribution of 32 GWAS-identified common variants to severe obesity in European adults referred for bariatric surgery. *PLoS One* **8**, e70735, (2013).

150 Hong, J. *et al.* Genetic susceptibility, birth weight and obesity risk in young Chinese. *Int J Obes (Lond)* **37**, 673-677, (2013).

151 Xi, B. *et al.* Study of 11 BMI-associated loci identified in GWAS for associations with central obesity in the Chinese children. *PLoS One* **8**, e56472, (2013).

152 Ruigrok, Y. M., Rinkel, G. J. & Wijmenga, C. The versican gene and the risk of intracranial aneurysms. *Stroke* **37**, 2372-2374, (2006).

153 Gudbjartsson, D. F. *et al.* A sequence variant in ZFHX3 on 16q22 associates with atrial fibrillation and ischemic stroke. *Nat Genet* **41**, 876-878, (2009).

154 Traylor, M. *et al.* Genetic risk factors for ischaemic stroke and its subtypes (the METASTROKE collaboration): a meta-analysis of genome-wide association studies. *Lancet Neurol* **11**, 951-962, (2012).

155 Holliday, E. G. *et al.* Common variants at 6p21.1 are associated with large artery atherosclerotic stroke. *Nat Genet* **44**, 1147-1151, (2012).

156 Sladek, R. *et al.* A genome-wide association study identifies novel risk loci for type 2 diabetes. *Nature* **445**, 881-885, (2007).

157 Wu, Y. *et al.* Common variants in CDKAL1, CDKN2A/B, IGF2BP2, SLC30A8, and HHEX/IDE genes are associated with type 2 diabetes and impaired fasting glucose in a Chinese Han population. *Diabetes* **57**, 2834-2842, (2008).

158 Unoki, H. *et al.* SNPs in KCNQ1 are associated with susceptibility to type 2 diabetes in East Asian and European populations. *Nat Genet* **40**, 1098-1102, (2008).

159 Rung, J. *et al.* Genetic variant near IRS1 is associated with type 2 diabetes, insulin resistance and hyperinsulinemia. *Nat Genet* **41**, 1110-1115, (2009).

160 Takeuchi, F. *et al.* Confirmation of multiple risk Loci and genetic impacts by a genome-wide association study of type 2 diabetes in the Japanese population. *Diabetes* **58**, 1690-1699, (2009).

161 Timpson, N. J. *et al.* Adiposity-related heterogeneity in patterns of type 2 diabetes susceptibility observed in genome-wide association data. *Diabetes* **58**, 505-510, (2009).

162 Shu, X. O. *et al.* Identification of new genetic risk variants for type 2 diabetes. *PLoS Genet* **6**, e1001127, (2010).

163 Tsai, F. J. *et al.* A genome-wide association study identifies susceptibility variants for type 2 diabetes in Han Chinese. *PLoS Genet* **6**, e1000847, (2010).

164 Han, X. *et al.* Implication of genetic variants near SLC30A8, HHEX, CDKAL1, CDKN2A/B, IGF2BP2, FTO, TCF2, KCNQ1, and WFS1 in type 2 diabetes in a Chinese population. *BMC Med Genet* **11**, 81, (2010).

165 Xu, M. *et al.* Combined effects of 19 common variations on type 2 diabetes in Chinese: results from two community-based studies. *PLoS One* **5**, e14022, (2010).

166 Morris, A. P. *et al.* Large-scale association analysis provides insights into the genetic architecture and pathophysiology of type 2 diabetes. *Nat Genet* **44**, 981-990, (2012).

167 Saxena, R. *et al.* Large-scale gene-centric meta-analysis across 39 studies identifies type 2 diabetes loci. *Am J Hum Genet* **90**, 410-425, (2012).

168 Villegas, R. *et al.* Joint effect of genetic and lifestyle risk factors on type 2 diabetes risk among Chinese men and women. *PLoS One* **7**, e49464, (2012).

169 Lu, F. *et al.* Genetic variants on chromosome 6p21.1 and 6p22.3 are associated with type 2 diabetes risk: a case-control study in Han Chinese. *J Hum Genet* **57**, 320-325, (2012).

170 Li, H. *et al.* A genome-wide association study identifies GRK5 and RASGRP1 as type 2 diabetes loci in Chinese Hans. *Diabetes* **62**, 291-298, (2013).

171 Kuo, J. Z. *et al.* Trans-ethnic fine mapping identifies a novel independent locus at the 3' end of CDKAL1 and novel variants of several susceptibility loci for type 2 diabetes in a Han Chinese population. *Diabetologia* **56**, 2619-2628, (2013).

172 Zhang, B. C., Li, W. M., Zhu, M. Y. & Xu, Y. W. Association of TCF7L2 gene polymorphisms with type 2 diabetes mellitus in Han Chinese population: a meta-analysis. *Gene* **512**, 76-81, (2013).

173 Ma, R. C. *et al.* Familial young-onset diabetes, pre-diabetes and cardiovascular disease are associated with genetic variants of DACH1 in Chinese. *PLoS One* **9**, e84770, (2014).

174 Replication, D. I. G. *et al.* Genome-wide trans-ancestry meta-analysis provides insight into the genetic architecture of type 2 diabetes susceptibility. *Nat Genet* **46**, 234-244, (2014).

175 Zhu, J. *et al.* Association of genetic predisposition to obesity with type 2 diabetes risk in Han Chinese individuals. *Diabetologia* **57**, 1830-1833, (2014).

176 Hara, K. *et al.* Genome-wide association study identifies three novel loci for type 2 diabetes. *Hum Mol Genet* **23**, 239-246, (2014).

177 Harold, D. *et al.* Genome-wide association study identifies variants at CLU and PICALM associated with Alzheimer's disease. *Nat Genet* **41**, 1088-1093, (2009).

178 Ferreira, M. A. *et al.* Collaborative genome-wide association analysis supports a role for ANK3 and CACNA1C in bipolar disorder. *Nat Genet* **40**, 1056-1058, (2008).

179 Cichon, S. *et al.* Genome-wide association study identifies genetic variation in neurocan as a susceptibility factor for bipolar disorder. *Am J Hum Genet* **88**, 372-381, (2011).

180 Hor, H. *et al.* Genome-wide association study identifies new HLA class II haplotypes strongly protective against narcolepsy. *Nat Genet* **42**, 786-789, (2010).

181 Kornum, B. R. *et al.* Common variants in P2RY11 are associated with narcolepsy. *Nat Genet* **43**, 66-71, (2011).

182 Satake, W. *et al.* Genome-wide association study identifies common variants at four loci as genetic risk factors for Parkinson's disease. *Nat Genet* **41**, 1303-1307, (2009).

183 Simon-Sanchez, J. *et al.* Genome-wide association study reveals genetic risk underlying Parkinson's disease. *Nat Genet* **41**, 1308-1312, (2009).

184 Edwards, T. L. *et al.* Genome-wide association study confirms SNPs in SNCA and the MAPT region as common risk factors for Parkinson disease. *Ann Hum Genet* **74**, 97-109, (2010).

185 Chang, X. L. *et al.* Association of GWAS loci with PD in China. *Am J Med Genet B Neuropsychiatr Genet* **156B**, 334-339, (2011).

186 Do, C. B. *et al.* Web-based genome-wide association study identifies two novel loci and a substantial genetic component for Parkinson's disease. *PLoS Genet* **7**, e1002141, (2011).

187 Pankratz, N. *et al.* Meta-analysis of Parkinson's disease: identification of a novel locus, RIT2. *Ann Neurol* **71**, 370-384, (2012).

188 Hill-Burns, E. M. *et al.* Identification of a novel Parkinson's disease locus via stratified genome-wide association study. *BMC Genomics* **15**, 118, (2014).

189 Lill, C. M. *et al.* Comprehensive research synopsis and systematic meta-analyses in Parkinson's disease genetics: The PDGene database. *PLoS Genet* **8**, e1002548, (2012).

190 Stefansson, H. *et al.* Common variants conferring risk of schizophrenia. *Nature* **460**, 744-747, (2009).

191 Shi, Y. *et al.* Common variants on 8p12 and 1q24.2 confer risk of schizophrenia. *Nat Genet* **43**, 1224-1227, (2011).

192 Yue, W. H. *et al.* Genome-wide association study identifies a susceptibility locus for schizophrenia in Han Chinese at 11p11.2. *Nat Genet* **43**, 1228-1231, (2011).

193 Schizophrenia Psychiatric Genome-Wide Association Study Consortium. Genome-wide association study identifies five new schizophrenia loci. *Nat Genet* **43**, 969-976, (2011).

194 Ripke, S. *et al.* Genome-wide association analysis identifies 13 new risk loci for schizophrenia. *Nat Genet* **45**, 1150-1159, (2013).

195 Holloway, J. W., Barton, S. J., Holgate, S. T., Rose-Zerilli, M. J. & Sayers, I. The role of LTA4H and ALOX5AP polymorphism in asthma and allergy susceptibility. *Allergy* **63**, 1046-1053, (2008).

196 Himes, B. E. *et al.* Genome-wide association analysis identifies PDE4D as an asthma-susceptibility gene. *Am J Hum Genet* **84**, 581-593, (2009).

197 Moffatt, M. F. *et al.* A large-scale, consortium-based genomewide association study of asthma. *N Engl J Med* **363**, 1211-1221, (2010).

198 Li, X. *et al.* Genome-wide association study of asthma identifies RAD50-IL13 and HLA-DR/DQ regions. *J Allergy Clin Immunol* **125**, 328-335 e311, (2010).

199 Ferreira, M. A. *et al.* Identification of IL6R and chromosome 11q13.5 as risk loci for asthma. *Lancet* **378**, 1006-1014, (2011).

200 Ramasamy, A. *et al.* Genome-wide association studies of asthma in population-based cohorts confirm known and suggested loci and identify an additional association near HLA. *PLoS One* **7**, e44008, (2012).

201 Wan, Y. I. *et al.* Genome-wide association study to identify genetic determinants of severe asthma. *Thorax* **67**, 762-768, (2012).

202 Li, X. *et al.* Genome-wide association studies of asthma indicate opposite immunopathogenesis direction from autoimmune diseases. *J Allergy Clin Immunol* **130**, 861-868 e867, (2012).

203 Ferreira, M. A. *et al.* Genome-wide association analysis identifies 11 risk variants associated with the asthma with hay fever phenotype. *J Allergy Clin Immunol* **133**, 1564-1571, (2014).

204 Liu, Y. *et al.* A genome-wide association study of psoriasis and psoriatic arthritis identifies new disease loci. *PLoS Genet* **4**, e1000041, (2008).

205 Nair, R. P. *et al.* Genome-wide scan reveals association of psoriasis with IL-23 and NF-kappaB pathways. *Nat Genet* **41**, 199-204, (2009).

206 Zhang, X. J. *et al.* Psoriasis genome-wide association study identifies susceptibility variants within LCE gene cluster at 1q21. *Nat Genet* **41**, 205-210, (2009).

207 Sun, L. D. *et al.* Association analyses identify six new psoriasis susceptibility loci in the Chinese population. *Nat Genet* **42**, 1005-1009, (2010).

208 Genetic Analysis of Psoriasis, C. *et al.* A genome-wide association study identifies new psoriasis susceptibility loci and an interaction between HLA-C and ERAP1. *Nat Genet* **42**, 985-990, (2010).

209 Stuart, P. E. *et al.* Genome-wide association analysis identifies three psoriasis susceptibility loci. *Nat Genet* **42**, 1000-1004, (2010).

210 Ellinghaus, E. *et al.* Genome-wide association study identifies a psoriasis susceptibility locus at TRAF3IP2. *Nat Genet* **42**, 991-995, (2010).

211 Tsoi, L. C. *et al.* Identification of 15 new psoriasis susceptibility loci highlights the role of innate immunity. *Nat Genet* **44**, 1341-1348, (2012).

212 Li, Y. *et al.* Association analyses identifying two common susceptibility loci shared by psoriasis and systemic lupus erythematosus in the Chinese Han population. *J Med Genet* **50**, 812-818, (2013).

213 Yin, X. *et al.* Genome-wide meta-analysis identifies multiple novel associations and ethnic heterogeneity of psoriasis susceptibility. *Nat Commun* **6**, 6916, (2015).

214 Baurecht, H. *et al.* Genome-wide comparative analysis of atopic dermatitis and psoriasis gives insight into opposing genetic mechanisms. *Am J Hum Genet* **96**, 104-120, (2015).

215 Raychaudhuri, S. *et al.* Common variants at CD40 and other loci confer risk of rheumatoid arthritis. *Nat Genet* **40**, 1216-1223, (2008).

216 Orozco, G. *et al.* Combined effects of three independent SNPs greatly increase the risk estimate for RA at 6q23. *Hum Mol Genet* **18**, 2693-2699, (2009).

217 Kochi, Y. *et al.* A regulatory variant in CCR6 is associated with rheumatoid arthritis susceptibility. *Nat Genet* **42**, 515-519, (2010).

218 Stahl, E. A. *et al.* Genome-wide association study meta-analysis identifies seven new rheumatoid arthritis risk loci. *Nat Genet* **42**, 508-514, (2010).

219 Jiang, L. *et al.* Novel risk loci for rheumatoid arthritis in Han Chinese and congruence with risk variants in Europeans. *Arthritis Rheumatol* **66**, 1121-1132, (2014).

220 Okada, Y. *et al.* Genetics of rheumatoid arthritis contributes to biology and drug discovery. *Nature* **506**, 376-381, (2014).

221 Hom, G. *et al.* Association of systemic lupus erythematosus with C8orf13-BLK and ITGAM-ITGAX. *N Engl J Med* **358**, 900-909, (2008).

222 International Consortium for Systemic Lupus Erythematosus, G. *et al.* Genome-wide association scan in women with systemic lupus erythematosus identifies susceptibility variants in ITGAM, PXK, KIAA1542 and other loci. *Nat Genet* **40**, 204-210, (2008).

223 Sigurdsson, S. *et al.* Comprehensive evaluation of the genetic variants of interferon regulatory factor 5 (IRF5) reveals a novel 5 bp length polymorphism as strong risk factor for systemic lupus erythematosus. *Hum Mol Genet* **17**, 872-881, (2008).

224 Kozyrev, S. V. *et al.* Functional variants in the B-cell gene BANK1 are associated with systemic lupus erythematosus. *Nat Genet* **40**, 211-216, (2008).

225 Musone, S. L. *et al.* Multiple polymorphisms in the TNFAIP3 region are independently associated with systemic lupus erythematosus. *Nat Genet* **40**, 1062-1064, (2008).

226 Graham, R. R. *et al.* Genetic variants near TNFAIP3 on 6q23 are associated with systemic lupus erythematosus. *Nat Genet* **40**, 1059-1061, (2008).

227 Han, J. W. *et al.* Genome-wide association study in a Chinese Han population identifies nine new susceptibility loci for systemic lupus erythematosus. *Nat Genet* **41**, 1234-1237, (2009).

228 Abelson, A. K. *et al.* STAT4 associates with systemic lupus erythematosus through two independent effects that correlate with gene expression and act additively with IRF5 to increase risk. *Ann Rheum Dis* **68**, 1746-1753, (2009).

229 Gateva, V. *et al.* A large-scale replication study identifies TNIP1, PRDM1, JAZF1, UHRF1BP1 and IL10 as risk loci for systemic lupus erythematosus. *Nat Genet* **41**, 1228-1233, (2009).

230 Zhang, Z. *et al.* Polymorphisms at 16p13 are associated with systemic lupus erythematosus in the Chinese population. *J Med Genet* **48**, 69-72, (2011).

231 Yang, W. *et al.* Meta-analysis followed by replication identifies loci in or near CDKN1B, TET3, CD80, DRAM1, and ARID5B as associated with systemic lupus erythematosus in Asians. *Am J Hum Genet* **92**, 41-51, (2013).

232 Martin, J. E. *et al.* A systemic sclerosis and systemic lupus erythematosus pan-meta-GWAS reveals new shared susceptibility loci. *Hum Mol Genet* **22**, 4021-4029, (2013).

233 Arakawa, S. *et al.* Genome-wide association study identifies two susceptibility loci for exudative age-related macular degeneration in the Japanese population. *Nat Genet* **43**, 1001-1004, (2011).

234 Yu, Y. *et al.* Common variants near FRK/COL10A1 and VEGFA are associated with advanced age-related macular degeneration. *Hum Mol Genet* **20**, 3699-3709, (2011).

235 Cipriani, V. *et al.* Genome-wide association study of age-related macular degeneration identifies associated variants in the TNXB-FKBPL-NOTCH4 region of chromosome 6p21.3. *Hum Mol Genet* **21**, 4138-4150, (2012).

236 Fritsche, L. G. *et al.* Seven new loci associated with age-related macular degeneration. *Nat Genet* **45**, 433-439, 439e431-432, (2013).

237 Vithana, E. N. *et al.* Genome-wide association analyses identify three new susceptibility loci for primary angle closure glaucoma. *Nat Genet* **44**, 1142-1146, (2012).

238 Chen, Y. *et al.* Common variants near ABCA1 and in PMM2 are associated with primary open-angle glaucoma. *Nat Genet* **46**, 1115-1119, (2014).

239 Gharahkhani, P. *et al.* Common variants near ABCA1, AFAP1 and GMDS confer risk of primary open-angle glaucoma. *Nat Genet* **46**, 1120-1125, (2014).

240 Chen, Y. *et al.* Genetic Variants Associated With Different Risks for High Tension Glaucoma and Normal Tension Glaucoma in a Chinese Population. *Invest Ophthalmol Vis Sci* **56**, 2595-2600, (2015).

241 Anttila, V. *et al.* Genome-wide meta-analysis identifies new susceptibility loci for migraine. *Nat Genet* **45**, 912-917, (2013).

242 Shi, Y. *et al.* A genome-wide association study identifies two new cervical cancer susceptibility loci at 4q12 and 17q12. *Nat Genet* **45**, 918-922, (2013).

243 Abnet, C. C. *et al.* A shared susceptibility locus in PLCE1 at 10q23 for gastric adenocarcinoma and esophageal squamous cell carcinoma. *Nat Genet* **42**, 764-767, (2010).

244 Wu, C. *et al.* Genome-wide association study identifies three new susceptibility loci for esophageal squamous-cell carcinoma in Chinese populations. *Nat Genet* **43**, 679-684, (2011).

245 Wu, C. *et al.* Genome-wide association analyses of esophageal squamous cell carcinoma in Chinese identify multiple susceptibility loci and gene-environment interactions. *Nat Genet* **44**, 1090-1097, (2012).

246 Abnet, C. C. *et al.* Genotypic variants at 2q33 and risk of esophageal squamous cell carcinoma in China: a meta-analysis of genome-wide association studies. *Hum Mol Genet* **21**, 2132-2141, (2012).

247 Levine, D. M. *et al.* A genome-wide association study identifies new susceptibility loci for esophageal adenocarcinoma and Barrett's esophagus. *Nat Genet* **45**, 1487-1493, (2013).

248 Shi, J. *et al.* Leukocyte telomere length-related genetic variants in 1p34.2 and 14q21 loci contribute to the risk of esophageal squamous cell carcinoma. *Int J Cancer* **132**, 2799-2807, (2013).

249 Chuang, S. C. *et al.* Polymorphism at the mucin-like protocadherin gene influences susceptibility to gallstone disease. *Clin Chim Acta* **412**, 2089-2093, (2011).

250 Li, S. *et al.* GWAS identifies novel susceptibility loci on 6p21.32 and 21q21.3 for hepatocellular carcinoma in chronic hepatitis B virus carriers. *PLoS Genet* **8**, e1002791, (2012).

251 Jiang, D. K. *et al.* Genetic variants in STAT4 and HLA-DQ genes confer risk of hepatitis B virus-related hepatocellular carcinoma. *Nat Genet* **45**, 72-75, (2013).

252 Jou, Y. S. *et al.* Association of an EGFR intron 1 SNP with never-smoking female lung adenocarcinoma patients. *Lung Cancer* **64**, 251-256, (2009).

253 Lan, Q. *et al.* Genome-wide association analysis identifies new lung cancer susceptibility loci in never-smoking women in Asia. *Nat Genet* **44**, 1330-1335, (2012).

254 Tse, K. P. *et al.* Genome-wide association study reveals multiple nasopharyngeal carcinoma-associated loci within the HLA region at chromosome 6p21.3. *Am J Hum Genet* **85**, 194-203, (2009).

255 Bei, J. X. *et al.* A genome-wide association study of nasopharyngeal carcinoma identifies three new susceptibility loci. *Nat Genet* **42**, 599-603, (2010).

256 Tang, M. *et al.* The principal genetic determinants for nasopharyngeal carcinoma in China involve the HLA class I antigen recognition groove. *PLoS Genet* **8**, e1003103, (2012).

257 Chen, Z. J. *et al.* Genome-wide association study identifies susceptibility loci for polycystic ovary syndrome on chromosome 2p16.3, 2p21 and 9q33.3. *Nat Genet* **43**, 55-59, (2011).

258 Shi, Y. *et al.* Genome-wide association study identifies eight new risk loci for polycystic ovary syndrome. *Nat Genet* **44**, 1020-1025, (2012).

259 Li, Y. *et al.* A genome-wide association study in Han Chinese identifies a susceptibility locus for primary Sjogren's syndrome at 7q11.23. *Nat Genet* **45**, 1361-1365, (2013).

260 Shi, Y. *et al.* A genome-wide association study identifies new susceptibility loci for non-cardia gastric cancer at 3q13.31 and 5p13.1. *Nat Genet* **43**, 1215-1218, (2011).

261 Hu, Y. *et al.* Two DNA repair gene polymorphisms on the risk of gastrointestinal cancers: a meta-analysis. *Tumour Biol* **35**, 1715-1725, (2014).

262 Chang, S. C. *et al.* Single nucleotide polymorphisms of one-carbon metabolism and cancers of the esophagus, stomach, and liver in a Chinese population. *PLoS One* **9**, e109235, (2014).

263 Yao, F. *et al.* Role of IL-17F T7488C polymorphism in carcinogenesis: a meta-analysis. *Tumour Biol* **35**, 9061-9068, (2014).

264 Wang, K., Xu, L., Pan, L., Xu, K. & Li, G. The functional BRCA1 rs799917 genetic polymorphism is associated with gastric cancer risk in a Chinese Han population. *Tumour Biol* **36**, 393-397, (2015).

265 Hu, N. *et al.* Genome-wide association study of gastric adenocarcinoma in Asia: a comparison of associations between cardia and non-cardia tumours. *Gut* **65**, 1611-1618, (2016).

266 Chen, Y. *et al.* Functional variants regulating LGALS1 (Galectin 1) expression affect human susceptibility to influenza A(H7N9). *Sci Rep* **5**, 8517, (2015).

267 Cheng, Z. *et al.* Identification of TMPRSS2 as a Susceptibility Gene for Severe 2009 Pandemic A(H1N1) Influenza and A(H7N9) Influenza. *J Infect Dis* **212**, 1214-1221, (2015).

268 To, K. K. W. *et al.* Surfactant protein B gene polymorphism is associated with severe influenza. *Chest* **145**, 1237-1243, (2014).

269 Wong, W. L. *et al.* Global prevalence of age-related macular degeneration and disease burden projection for 2020 and 2040: a systematic review and meta-analysis. *Lancet Glob Health* **2**, e106-116, (2014).

270 Hammond, C. J. *et al.* Genetic influence on early age-related maculopathy: a twin study. *Ophthalmology* **109**, 730-736, (2002).

271 Seddon, J. M., Cote, J., Page, W. F., Aggen, S. H. & Neale, M. C. The US twin study of age-related macular degeneration: relative roles of genetic and environmental influences. *Arch Ophthalmol* **123**, 321-327, (2005).

272 Alzheimer's Disease International. World Alzheimer Report 2015 -The Global Impact of Dementia: An analysis of prevalence, incidence, cost and trends. https://www.alz.co.uk/research/WorldAlzheimerReport2015.pdf (2015).

273 Gatz, M. *et al.* Role of genes and environments for explaining Alzheimer disease. *Arch Gen Psychiatry* **63**, 168-174, (2006).

274 To, T. *et al.* Global asthma prevalence in adults: findings from the cross-sectional world health survey. *BMC Public Health* **12**, 204, (2012).

275 Zahran, H. S. & Bailey, C. Factors associated with asthma prevalence among racial and ethnic groups--United States, 2009-2010 behavioral risk factor surveillance system. *J Asthma* **50**, 583-589, (2013).

276 Duffy, D. L., Martin, N. G., Battistutta, D., Hopper, J. L. & Mathews, J. D. Genetics of asthma and hay fever in Australian twins. *Am Rev Respir Dis* **142**, 1351-1358, (1990).

277 Tan, H., Walker, M., Gagnon, F. & Wen, S. W. The estimation of heritability for twin data based on concordances of sex and disease. *Chronic Dis Can* **26**, 9-12, (2005).

278 Ober, C. & Yao, T. C. The genetics of asthma and allergic disease: a 21st century perspective. *Immunol Rev* **242**, 10-30, (2011).

279 Merikangas, K. R. *et al.* Prevalence and correlates of bipolar spectrum disorder in the world mental health survey initiative. *Arch Gen Psychiatry* **68**, 241-251, (2011).

280 McGuffin, P. *et al.* The heritability of bipolar affective disorder and the genetic relationship to unipolar depression. *Arch Gen Psychiatry* **60**, 497-502, (2003).

281 Kieseppa, T., Partonen, T., Haukka, J., Kaprio, J. & Lonnqvist, J. High concordance of bipolar I disorder in a nationwide sample of twins. *Am J Psychiatry* **161**, 1814-1821, (2004).

282 Lichtenstein, P. *et al.* Common genetic determinants of schizophrenia and bipolar disorder in Swedish families: a population-based study. *Lancet* **373**, 234-239, (2009).

283 Bray, F., Ren, J. S., Masuyer, E. & Ferlay, J. Global estimates of cancer prevalence for 27 sites in the adult population in 2008. *Int J Cancer* **132**, 1133-1145, (2013).

284 Lichtenstein, P. *et al.* Environmental and heritable factors in the causation of cancer--analyses of cohorts of twins from Sweden, Denmark, and Finland. *N Engl J Med* **343**, 78-85, (2000).

285 Sapkota, Y. Germline DNA variations in breast cancer predisposition and prognosis: a systematic review of the literature. *Cytogenet Genome Res* **144**, 77-91, (2014).

286 Vernooij, M. W. *et al.* Incidental findings on brain MRI in the general population. *N Engl J Med* **357**, 1821-1828, (2007).

287 Chan, D. Y. *et al.* Screening for intracranial aneurysms? Prevalence of unruptured intracranial aneurysms in Hong Kong Chinese. *J Neurosurg* **124**, 1245-1249, (2016).

288 Astradsson, A. & Astrup, J. An intracranial aneurysm in one identical twin, but no aneurysm in the other. *Br J Neurosurg* **15**, 168-171, (2001).

289 Magnusson, P. K., Lichtenstein, P. & Gyllensten, U. B. Heritability of cervical tumours. *Int J Cancer* **88**, 698-701, (2000).

290 Department of Health, Hong Kong Special Administrative Region. Population health survey 2003/04.

http://www.chp.gov.hk/files/pdf/report_on_population_health_survey_2003_2004_en.pdf (2004).

291 Go, A. S. *et al.* Heart disease and stroke statistics--2013 update: a report from the American Heart Association. *Circulation* **127**, e6-e245, (2013).

292 Townsend, N., Bhatnagar, P., Wilkins, E., Wickramasinghe, K. & Rayner, M. Cardiovascular disease statisics 2015. *British Heart Foundation*. https://www.bhf.org.uk/publications/statistics/cvd-stats-2015 (2015).

293 Fischer, M. *et al.* Distinct heritable patterns of angiographic coronary artery disease in families with myocardial infarction. *Circulation* **111**, 855-862, (2005).

294 Economou, M., Zambeli, E. & Michopoulos, S. Incidence and prevalence of Crohn's disease and its etiological influences. http://www.annalsgastro.gr/index.php/annalsgastro/article/view/743 (2009).

295 Zheng, J. J., Zhu, X. S., Huangfu, Z., Shi, X. H. & Guo, Z. R. Prevalence and incidence rates of Crohn's disease in mainland China: a meta-analysis of 55 years of research. *J Dig Dis* **11**, 161-166, (2010).

296 Tysk, C., Lindberg, E., Jarnerot, G. & Floderus-Myrhed, B. Ulcerative colitis and Crohn's disease in an unselected population of monozygotic and dizygotic twins. A study of heritability and the influence of smoking. *Gut* **29**, 990-996, (1988).

297 Mucci, L. A. *et al.* Familial Risk and Heritability of Cancer Among Twins in Nordic Countries. *JAMA* **315**, 68-76, (2016).

298 Lu, Y. *et al.* Most common 'sporadic' cancers have a significant germline genetic component. *Hum Mol Genet* **23**, 6112-6118, (2014).

299 Xu, Q. *et al.* Prevalences of and risk factors for biliary stones and gallbladder polyps in a large Chinese population. *HPB (Oxford)* **14**, 373-381, (2012).

300 Nakeeb, A. *et al.* Gallstones: genetics versus environment. *Ann Surg* **235**, 842-849, (2002).

301 Katsika, D. *et al.* Genetic and environmental influences on symptomatic gallstone disease: a Swedish study of 43,141 twin pairs. *Hepatology* **41**, 1138-1143, (2005).

302 Tham, Y. C. *et al.* Global prevalence of glaucoma and projections of glaucoma burden through 2040: a systematic review and meta-analysis. *Ophthalmology* **121**, 2081-2090, (2014).

303 Chang, T. C. *et al.* Determinants and heritability of intraocular pressure and cup-to-disc ratio in a defined older population. *Ophthalmology* **112**, 1186-1191, (2005).

304 Pu, J. K. S., Ng, G. K. B., Leung, G. K. K. & Wong, C.-K. One-year review of the incidence of brain tumours in Hong Kong Chinese patients as part of the Hong Kong Brain and Spinal Tumours Registry. *Surgical Practice* **16**, 133-136, (2012).

305 Crocetti, E. *et al.* Epidemiology of glial and non-glial brain tumours in Europe. *Eur J Cancer* **48**, 1532-1542, (2012).

306 Kinnersley, B. *et al.* Quantifying the heritability of glioma using genome-wide complex trait analysis. *Sci Rep* **5**, 17267, (2015).

307 World Health Organization. *Global health observatory data-raised cholesterol*. http://www.who.int/gho/ncd/risk_factors/cholesterol_text/en/ (2008).

308 Ni, W.-Q. *et al.* Serum lipids and associated factors of dyslipidemia in the adult population in Shenzhen. *Lipids in Health and Disease* **14**, 71, (2015).

309 Chen, C. J. *et al.* Genetic variance and heritability of serum cholesterol and triglycerides among Chinese twin neonates. *Acta Genet Med Gemellol (Roma)* **39**, 123-131, (1990).

310 Department of Health, Hong Kong Special Administrative Region. Hypertension is preventable and treatable. http://www.dh.gov.hk/english/press/2013/130402-2.html (2013).

311 Dreisbach, D. W. Epidemiology of hypertension. http://emedicine.medscape.com/article/1928048-overview#a2 (2014).

312 Luft, F. C. Twins in cardiovascular genetic research. *Hypertension* **37**, 350-356, (2001).

313 Agarwal, A., Williams, G. H. & Fisher, N. D. Genetics of human hypertension. *Trends Endocrinol Metab* **16**, 127-133, (2005).

314 Miller, M. *et al.* Triglycerides and cardiovascular disease. *A Scientific Statement From the American Heart Association*, (2011). doi:10.1161/CIR.0b013e3182160726.

315 Heller, D. A., de Faire, U., Pedersen, N. L., Dahlen, G. & McClearn, G. E. Genetic and environmental influences on serum lipid levels in twins. *N Engl J Med* **328**, 1150-1156, (1993).

316 Osther, P. J. S. in *Urolithiasis: Basic Science and Clinical Practice* (eds Jamsheer J. Talati, Hans-Goran Tiselius, David M. Albala, & Zhangqun Ye) 3-12 (Springer London, 2012).

317 Scales, C. D., Jr., Smith, A. C., Hanley, J. M., Saigal, C. S. & Urologic Diseases in America, P. Prevalence of kidney stones in the United States. *Eur Urol* **62**, 160-165, (2012).

318 Zeng, Q. & He, Y. Age-specific prevalence of kidney stones in Chinese urban inhabitants. *Urolithiasis* **41**, 91-93, (2013).

319 Goldfarb, D. S., Fischer, M. E., Keich, Y. & Goldberg, J. A twin study of genetic and dietary influences on nephrolithiasis: a report from the Vietnam Era Twin (VET) Registry. *Kidney Int* **67**, 1053-1061, (2005).

320 Meng, W. *et al.* [A study on the genetic epidemiology of hepatocellular carcinoma]. *Zhonghua Liu Xing Bing Xue Za Zhi* **23**, 438-440, (2002).

321 Hemminki, K., Lonnstedt, I., Vaittinen, P. & Lichtenstein, P. Estimation of genetic and environmental components in colorectal and lung cancer and melanoma. *Genet Epidemiol* **20**, 107-116, (2001).

322 Czene, K., Lichtenstein, P. & Hemminki, K. Environmental and heritable causes of cancer among 9.6 million individuals in the Swedish Family-Cancer Database. *Int J Cancer* **99**, 260-266, (2002).

323 Yang, I. A., Holloway, J. W. & Fong, K. M. Genetic susceptibility to lung cancer and co-morbidities. *J Thorac Dis* **5 Suppl 5**, S454-462, (2013).

324 Toh, C. K. *et al.* Never-smokers with lung cancer: epidemiologic evidence of a distinct disease entity. *Journal of clinical oncology : official journal of the American Society of Clinical Oncology* **24**, 2245-2251, (2006).

325 Lipton, R. B., Stewart, W. F., Diamond, S., Diamond, M. L. & Reed, M. Prevalence and burden of migraine in the United States: data from the American Migraine Study II. *Headache* **41**, 646-657, (2001).

326 Steiner, T. J. *et al.* The prevalence and disability burden of adult migraine in England and their relationships to age, gender and ethnicity. *Cephalalgia* **23**, 519-527, (2003).

327 Luo, N. *et al.* Prevalence and burden of headache disorders in two neighboring provinces of China. *J Clin Neurosci* **21**, 1750-1754, (2014).

328 Mulder, E. J. *et al.* Genetic and environmental influences on migraine: a twin study across six countries. *Twin Res* **6**, 422-431, (2003).

329 Xiao, J., Chen, L. H., Tu, Y. T., Deng, X. H. & Tao, J. Prevalence of myocardial infarction in patients with psoriasis in central China. *J Eur Acad Dermatol Venereol* **23**, 1311-1315, (2009).

330 Bhatnagar, P., Wickramasinghe, K., Williams, J., Rayner, M. & Townsend, N. The epidemiology of cardiovascular disease in the UK 2014. *Heart* **101**, 1182-1189, (2015).

331 Mozaffarian, D. *et al.* Heart disease and stroke statistics--2015 update: a report from the American Heart Association. *Circulation* **131**, e29-322, (2015).

332 Wing, Y. K. *et al.* The prevalence of narcolepsy among Chinese in Hong Kong. *Ann Neurol* **51**, 578-584, (2002).

333 Longstreth, W. T., Jr., Koepsell, T. D., Ton, T. G., Hendrickson, A. F. & van Belle, G. The epidemiology of narcolepsy. *Sleep* **30**, 13-26, (2007).

334 Chen, C. J. *et al.* Multiple risk factors of nasopharyngeal carcinoma: Epstein-Barr virus, malarial infection, cigarette smoking and familial tendency. *Anticancer Res* **10**, 547-553, (1990).

335 Berndt, S. I. *et al.* Genetic heritability of common non-Hodgkin lymphoma subtypes. in *American Society of Human Genetics 64th Annual Meeting* (San Diego, CA, 2014).

336 Ng, M. *et al.* Global, regional, and national prevalence of overweight and obesity in children and adults during 1980-2013: a systematic analysis for the Global Burden of Disease Study 2013. *Lancet* **384**, 766-781, (2014).

337 O'Rahilly, S. & Farooqi, I. S. Human obesity: a heritable neurobehavioral disorder that is highly sensitive to environmental conditions. *Diabetes* **57**, 2905-2910, (2008).

338 Schildkraut, J. M., Risch, N. & Thompson, W. D. Evaluating genetic association among ovarian, breast, and endometrial cancer: evidence for a breast/ovarian cancer relationship. *Am J Hum Genet* **45**, 521-529, (1989).

339 Woo, J., Lau, E., Ziea, E. & Chan, D. K. Prevalence of Parkinson's disease in a Chinese population. *Acta Neurol Scand* **109**, 228-231, (2004).

340 Pringsheim, T., Jette, N., Frolkis, A. & Steeves, T. D. The prevalence of Parkinson's disease: a systematic review and meta-analysis. *Mov Disord* **29**, 1583-1590, (2014).

341 Li, R. *et al.* Prevalence of polycystic ovary syndrome in women in China: a large community-based study. *Hum Reprod* **28**, 2562-2569, (2013).

342 Vink, J. M., Sadrzadeh, S., Lambalk, C. B. & Boomsma, D. I. Heritability of polycystic ovary syndrome in a Dutch twin-family study. *J Clin Endocrinol Metab* **91**, 2100-2104, (2006).

343 Liu, H. *et al.* Prevalence of primary biliary cirrhosis in adults referring hospital for annual health check-up in Southern China. *BMC Gastroenterol* **10**, 100, (2010).

344 Boonstra, K., Beuers, U. & Ponsioen, C. Y. Epidemiology of primary sclerosing cholangitis and primary biliary cirrhosis: a systematic review. *J Hepatol* **56**, 1181-1188, (2012).

345 Mells, G. F. Primary biliary cirrhosis: Family, genes, and bugs. *Clinical Liver Disease* **3**, 69-73, (2014).

346 Hjelmborg, J. B. *et al.* The heritability of prostate cancer in the Nordic Twin Study of Cancer. *Cancer Epidemiol Biomarkers Prev* **23**, 2303-2310, (2014).

347 Ding, X. *et al.* Prevalence of psoriasis in China: a population-based study in six cities. *Eur J Dermatol* **22**, 663-667, (2012).

348 Rachakonda, T. D., Schupp, C. W. & Armstrong, A. W. Psoriasis prevalence among adults in the United States. *J Am Acad Dermatol* **70**, 512-516, (2014).

349 World Health Organization. Global report on psoriasis. http://apps.who.int/iris/bitstream/10665/204417/1/9789241565189_eng.pdf (2016).

350 Lonnberg, A. S. *et al.* Heritability of psoriasis in a large twin sample. *Br J Dermatol* **169**, 412-416, (2013).

351 Helmick, C. G. *et al.* Estimates of the prevalence of arthritis and other rheumatic conditions in the United States: Part I. *Arthritis & Rheumatism* **58**, 15-25, (2008).

352 Li, R. *et al.* Epidemiology of eight common rheumatic diseases in China: a large-scale cross-sectional survey in Beijing. *Rheumatology (Oxford)* **51**, 721-729, (2012).

353 Frisell, T. *et al.* Familial risks and heritability of rheumatoid arthritis: role of rheumatoid factor/anti-citrullinated protein antibody status, number and type of affected relatives, sex, and age. *Arthritis Rheum* **65**, 2773-2782, (2013).

354 Long, J. *et al.* The prevalence of schizophrenia in mainland China: evidence from epidemiological surveys. *Acta Psychiatr Scand* **130**, 244-256, (2014).

355 Moreno-Kustner, B. *et al.* Prevalence of schizophrenia and related disorders in Malaga (Spain): results using multiple clinical databases. *Epidemiol Psychiatr Sci* **25**, 38-48, (2016).

356 Sullivan, P. F., Kendler, K. S. & Neale, M. C. Schizophrenia as a complex trait: evidence from a meta-analysis of twin studies. *Arch Gen Psychiatry* **60**, 1187-1192, (2003).

357 Zhang, N. Z. *et al.* Prevalence of primary Sjogren's syndrome in China. *J Rheumatol* **22**, 659-661, (1995).

358 Kuo, C. F. *et al.* Familial Risk of Sjogren's Syndrome and Co-aggregation of Autoimmune Diseases in Affected Families: A Nationwide Population Study. *Arthritis Rheumatol* **67**, 1904-1912, (2015).

359 Liu, M. *et al.* Stroke in China: epidemiology, prevention, and management strategies. *Lancet Neurol* **6**, 456-464, (2007).

360 Centers for Disease, C. & Prevention. Prevalence of stroke--United States, 2006-2010. *MMWR Morb Mortal Wkly Rep* **61**, 379-382, (2012).

361 Bevan, S. *et al.* Genetic heritability of ischemic stroke and the contribution of previously reported candidate gene and genomewide associations. *Stroke* **43**, 3161-3167, (2012).

362 Mok, C. C. Epidemiology and survival of systemic lupus erythematosus in Hong Kong Chinese. *Lupus* **20**, 767-771, (2011).

363 Rees, F. *et al.* The incidence and prevalence of systemic lupus erythematosus in the UK, 1999-2012. *Ann Rheum Dis* **75**, 136-141, (2016).

364 Harley, I. T., Kaufman, K. M., Langefeld, C. D., Harley, J. B. & Kelly, J. A. Genetic susceptibility to SLE: new insights from fine mapping and genome-wide association studies. *Nat Rev Genet* **10**, 285-290, (2009).

365 International Diabetes Federation. *IDF diabetes atlas*. 6 edn, (International Diabetes Federation, 2013).

366 Centres for Disease Control and Prevention. National diabetes statistics report: estimates of diabetes and its burden in the United States. https://www.cdc.gov/diabetes/pubs/statsreport14/national-diabetes-report-web.pdf (2014).

367 Poulsen, P., Kyvik, K. O., Vaag, A. & Beck-Nielsen, H. Heritability of type II (non-insulin-dependent) diabetes mellitus and abnormal glucose tolerance--a population-based twin study. *Diabetologia* **42**, 139-145, (1999).

368 Chow, D. K. *et al.* Long-term follow-up of ulcerative colitis in the Chinese population. *Am J Gastroenterol* **104**, 647-654, (2009).

369 Loftus, E. V., Jr. Clinical epidemiology of inflammatory bowel disease: Incidence, prevalence, and environmental influences. *Gastroenterology* **126**, 1504-1517, (2004).

370 Chen, G. B. *et al.* Estimation and partitioning of (co)heritability of inflammatory bowel disease from GWAS and immunochip data. *Hum Mol Genet* **23**, 4710-4720, (2014).

371 Armstrong, R. A. & Mousavi, M. Overview of Risk Factors for Age-Related Macular Degeneration (AMD). *J Stem Cells* **10**, 171-191, (2015).

372 Seshadri, S. *et al.* Lifetime risk of dementia and Alzheimer's disease. The impact of mortality on risk estimates in the Framingham Study. *Neurology* **49**, 1498-1504, (1997).

373 To, T., Wang, C., Guan, J., McLimont, S. & Gershon, A. S. What is the lifetime risk of physician-diagnosed asthma in Ontario, Canada? *Am J Respir Crit Care Med* **181**, 337-343, (2010).

374 American Cancer Society. Cancer facts and figures 2015. https://www.cancer.org/content/dam/cancer-org/research/cancer-facts-and-statistics/annual-cancer-facts-and-figures/2015/cancer-facts-and-figures-2015.pdf (2015).

375 Hong Kong Hospital Authority. Hong Kong cancer registry. https://www3.ha.org.hk/cancereg/facts.html (2013).

376 Lloyd-Jones, D. M., Larson, M. G., Beiser, A. & Levy, D. Lifetime risk of developing coronary heart disease. *Lancet* **353**, 89-92, (1999).

377 Chuang, C. H. *et al.* Increasing incidence and lifetime risk of inflammatory bowel disease in Taiwan: a nationwide study in a low-endemic area 1998-2010. *Inflamm Bowel Dis* **19**, 2815-2819, (2013).

378 Chandana, S. R., Movva, S., Arora, M. & Singh, T. Primary brain tumors in adults. *Am Fam Physician* **77**, 1423-1430, (2008).

379 Pencina, M. J., D'Agostino, R. B., Beiser, A. S., Cobain, M. R. & Vasan, R. S. Estimating Lifetime Risk of Developing High Serum Total Cholesterol: Adjustment for Baseline Prevalence and Single-Occasion Measurements. *Am J Epidemiol* **165**, 464-472, (2007).

380 Lewington, S. *et al.* The Burden of Hypertension and Associated Risk for Cardiovascular Mortality in China. *JAMA Intern Med* **176**, 524-532, (2016).

381 Cancer Research UK. *Lifetime risk of cancer*. http://www.cancerresearchuk.org/health-professional/cancer-statistics/risk/lifetime-risk

382 Berry, J. D. *et al.* Lifetime risks of cardiovascular disease. *N Engl J Med* **366**, 321-329, (2012).

383 National Cancer Institute. *Surveillance, epidemiology, and end results (SEER) program, cancer stat facts: non-Hodgkin lymphoma*. https://seer.cancer.gov/statfacts/html/nhl.html (2016).

384 Elbaz, A. *et al.* Risk tables for parkinsonism and Parkinson's disease. *J Clin Epidemiol* **55**, 25-31, (2002).

385 Swanbeck, G. *et al.* Genetic counselling in psoriasis: empirical data on psoriasis among first-degree relatives of 3095 psoriatic probands. *Br J Dermatol* **137**, 939-942, (1997).

386 Crowson, C. S. *et al.* The lifetime risk of adult-onset rheumatoid arthritis and other inflammatory autoimmune rheumatic diseases. *Arthritis Rheum* **63**, 633-639, (2011).

387 Seshadri, S. & Wolf, P. A. Lifetime risk of stroke and dementia: current concepts, and estimates from the Framingham Study. *Lancet Neurol* **6**, 1106-1114, (2007).

388 Centers for Disease Control and Prevention. National diabetes statistics report: estimates of diabetes and its burden in the United States. https://www.cdc.gov/diabetes/pubs/statsreport14/national-diabetes-report-web.pdf
